# A Pilot Trial of a Sexual Health Counseling Intervention for HIV-Positive Gay and Bisexual Men Who Report Anal Sex without Condoms

**DOI:** 10.1371/journal.pone.0152762

**Published:** 2016-04-07

**Authors:** Trevor A. Hart, Natalie Stratton, Todd A. Coleman, Holly A. Wilson, Scott H. Simpson, Rick E. Julien, David Hoe, Bob Leahy, John Maxwell, Barry D. Adam

**Affiliations:** 1 Department of Psychology, Ryerson University, Toronto, Ontario, Canada; 2 Dalla Lana School of Public Health, University of Toronto, Toronto, Ontario, Canada; 3 AIDS Committee of Toronto, Toronto, Ontario, Canada; 4 Poz Prevention Working Group, Gay Men’s Sexual Health Alliance, Toronto, Ontario, Canada; 5 Department of Sociology, Anthropology and Criminology, University of Windsor, Windsor, Ontario, Canada; 6 Ontario HIV Treatment Network, Toronto, Ontario, Canada; Asociacion Civil Impacta Salud y Educacion, PERU

## Abstract

**Background:**

Even in the presence of promising biomedical treatment as prevention, HIV incidence among men who have sex with men has not always decreased. Counseling interventions, therefore, continue to play an important role in reducing HIV sexual transmission behaviors among gay and bisexual men and other men who have sex with men. The present study evaluated effects of a small-group counseling intervention on psychosocial outcomes and HIV sexual risk behavior.

**Method:**

HIV-positive (HIV+) peer counselors administered seven 2-hour counseling sessions to groups of 5 to 8 HIV+ gay and bisexual men. The intervention employed information provision, motivational interviewing, and behavioral skills building to reduce sexual transmission risk behaviors.

**Results:**

There was a significant reduction in condomless anal sex (CAS) with HIV-negative and unknown HIV-status partners, from 50.0% at baseline to 28.9% of the sample at 3-month follow-up. Findings were robust even when controlling for whether the participant had an undetectable viral load at baseline. Significant reductions were also found in the two secondary psychosocial outcomes, loneliness and sexual compulsivity.

**Conclusions:**

The findings provide preliminary evidence that this intervention may offer an efficient way of concurrently reducing CAS and mental health problems, such as sexual compulsivity and loneliness, for HIV+ gay and bisexual men.

**Trial Registration:**

ClinicalTrials.gov NCT02546271

## Introduction

HIV prevalence among gay, bisexual, and other men who have sex with men (GBM) in major cities continues to be high, with a 23.8% HIV prevalence among GBM in Toronto.[1] Among GBM there are elevated and rising rates of condomless anal sex (CAS) with partners of unknown or serodiscordant HIV status (HIV-negative men having CAS with HIV-positive [HIV+] men), as well as elevated syphilis rates.[2–4] Given that 1) HIV can transmit more efficiently during CAS than during condomless vaginal sex,[5] 2) HIV+ GBM are up to 3 times more likely than are HIV-negative GBM to have had CAS in the past 6 months,[6–8] and 3) most HIV behavioral risk reduction programs for GBM exclude or have low proportions of HIV+ men who have sex with men (MSM),[9] it is critical that HIV prevention counseling programs are developed that include HIV+ GBM. While recent data suggest that HIV medications may dramatically reduce HIV infectiousness,[10] mathematical modelling data indicate that condom use remains an important tool to keep HIV and STI incidence rates from rising among GBM.[4,11]

One factor that may account for the increasing HIV prevalence among GBM in Western countries is the rise of intentional CAS, which is colloquially termed in the gay community as barebacking.[6] The development of sub-groups of HIV+ GBM who bareback may partially account for a cascade of recent evidence showing elevated rates of CAS among HIV+ GBM with partners of unknown or negative HIV status.[12–25] The relative lack of behavioral interventions for HIV+ GBM is problematic because behavioral HIV prevention interventions including samples with more than 35% HIV+ GBM were not efficacious according to a meta-analysis.[26] In fact, another meta-analysis examining HIV prevention interventions delivered to HIV+ persons found that interventions were efficacious only when they did not focus on HIV+ GBM.[27] Even in the age of HIV antiretroviral medication treatments used for both HIV+ and HIV-negative persons to prevent HIV transmission[28,29] there is still an unmet need for empirically tested interventions to reduce sexual risk behavior among HIV+ GBM who engage in CAS with non-HIV+ partners, whether intentionally or spontaneously.

The empirically validated Information-Motivation-Behavioral Skills (IMB) model specifies that both HIV prevention information and motivation increase HIV risk reduction behavior directly as well as by improving behavioral skills, such as effective condom negotiation.[30] Our implementation model was built directly from the IMB theoretical model using interactive information provision, motivational interviewing, as well as role-plays and self-directed behavioral skills practice.[31–33] Motivational interviewing is a type of counseling designed to induce rapid, internally-motivated change by using the participants’ own change resources.[34] Counseling using this model has been successful in reducing HIV sexual transmission risk behavior among heterosexual populations.[35]

We hypothesized that this group-based program, called Gay Poz Sex (GPS), would result in a reduced prevalence of CAS at 3-month follow-up with partners who are HIV-negative or of unknown HIV serostatus. A secondary hypothesis is that GPS would lead to a reduced prevalence of CAS at 3-month follow-up with partners who are HIV+. The study also examined whether there would be reductions in psychosocial outcomes at 3-month follow-up, specifically depression, loneliness, fear of being sexually rejected for insisting on condom use, sexual compulsivity, and sensation seeking. Depression, loneliness, sexual compulsivity, and sexual sensation seeking, were considered due to their roles as risk factors for CAS[36–39] and the high prevalence of depression and loneliness experienced by many HIV+ GBM.[40,41] Lastly, we hypothesized that following the completion of the GPS intervention, participants would report an increased degree of self-efficacy to engage in sexual risk reduction behaviors. To support the ecological validity of this program, GPS is administered by paraprofessional HIV+ gay male facilitators at an AIDS service organization, which is a community-based organization providing psychosocial services in HIV care and prevention.

## Method

### Participants

**Fig 1:**
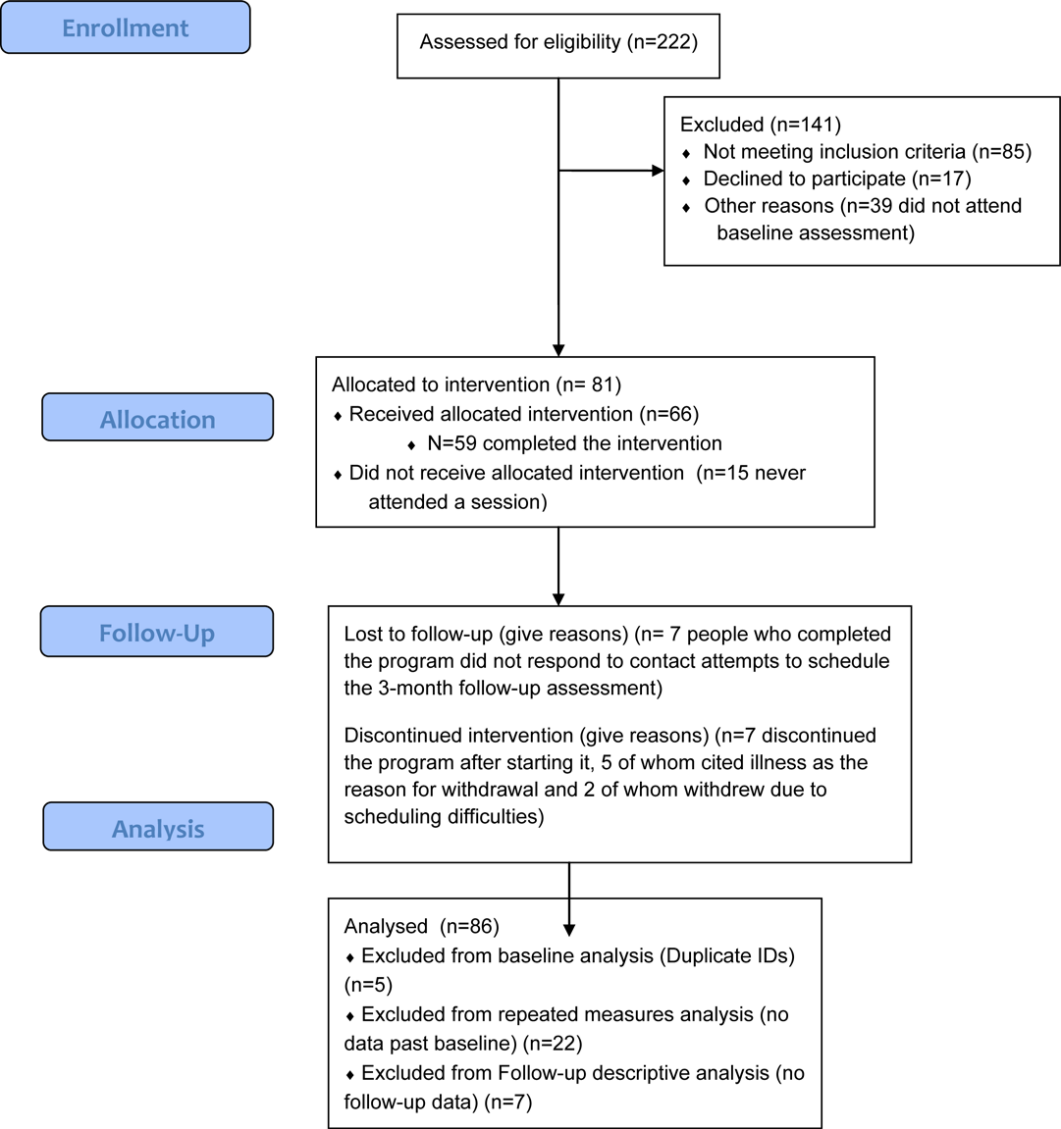
CONSORT diagram.

This study protocol was approved by the Research Ethics Board of Ryerson University (2007–176) and the University of Windsor. There were no protocol deviations or adverse events. All participants provided written informed consent at the outset of the study. Participants were recruited through posters placed at venues (e.g., bars, bathhouses) and community organizations (e.g., HIV/AIDS service organizations) within the Toronto metropolitan area, electronic advertisements on social media and websites targeting gay men, flyers distributed at local community events (i.e., Toronto Pride Street Festival), and the study’s website, http://gaypozsex.org/. In order to be eligible to participate in the GPS program, participants needed to 1) identify as male, 2) report engaging in CAS with another male during the past 3 months, 3) self-report an HIV+ status, 4) be over the age of 18 years old, and 5) be able to speak and read in English. The participant recruitment process is summarized in Fig 1. A total of 82 gay HIV+ men enrolled in the GPS program. Twenty-three participants did not complete the program because they did not present to the first session or dropped out of the GPS group. The final sample consisted of 59 men, of which 69% reported an undetectable viral load at baseline. Completers did not differ from non-completers in demographic variables, viral load detectability, depression, loneliness, sexual compulsivity, or in CAS outcome variables.

### Procedures

Interested participants contacted the study coordinator via telephone or email, in order to complete a brief telephone interview to determine whether they met eligibility criteria for this study. If eligible, participants were invited to Ryerson University for a 1-hour session during which they completed a computer-assisted self-interview questionnaire package. All participants provided written informed consent at the outset of the study. Subsequently, participants attended seven weekly 2-hour group sessions, led by 2 peer facilitators, who were HIV + gay men. The peer facilitators were paraprofessionals who were trained in the GPS protocol by a clinical psychologist. Each group consisted of 5–8 gay men and 2 facilitators. Sessions 1 and 2 comprise the informational component of the GPS program, focusing on the provision of information on topics related to HIV transmission, sexually transmitted infections, and the challenges of disclosing one’s HIV status. Sessions 3 through 5 focus on motivation by helping participants to identify their personal sexual health goals, and resolve any ambivalence between their personal goals and their current behavior. Sessions 6 and 7 provide behavioral skills, such as practicing asserting oneself in sexual situations. For a detailed description of the GPS program, refer to Hart et al., 2015.[42] Immediately and 3 months following the completion of the GPS program, participants were scheduled to attend a 1-hour session to complete the same questionnaire package. Participants received $30 and a list of community resources, including mental health or substance use counseling services, at the end of each assessment. The trial was registered after recruitment had been completed, as at the beginning of the trial it was not yet normative practice for pilot, non-randomized control trials to be registered. The trial began in March 2009 and the last follow-up was completed in April 2013. The trial registration number was NCT02546271 on clinicaltrials.gov; URL: https://clinicaltrials.gov/ct2/show/NCT02546271.

### Measures

#### Demographics

Sociodemographic characteristics recorded at baseline include age, ethnicity, employment status, educational attainment, annual income, and years since HIV diagnosis. Ethnic and sexual orientation categories were specified first by consultation with the Poz Prevention Working Group of the Ontario Gay Men’s Sexual Health Alliance. In addition, participants could select whether another category better represented their sexual orientation or ethnicity. The researchers were commissioned by the Poz Prevention Working Group to create an HIV prevention program for gay and bisexual men.

#### Depression

The Center for Epidemiologic Studies Depression Scale (CES-D)[43] consists of 20-items developed to assess depressive symptoms in the general population. Participants indicated on a 4-point Likert-type scale (ranging from 0 = rarely or none of the time to 3 = most or all of the time) the frequency of each symptom over the past week. Total scores range from 0 to 60, where higher total scores denote higher depressive symptomology. Total scores above 16 are indicative of clinically significant depressive symptomology. The CES-D demonstrates high internal consistency and moderate test-retest reliability.[43] In this study, the CES-D exhibited high internal consistency, with Cronbach’s α coefficients of 0.90, 0.93, and 0.91 at baseline, post-intervention, and 3-month follow-up, respectively.

#### Loneliness

To assess the extent to which participants felt lonely, the UCLA Loneliness Scale[44] was administered. Participants rated on a 4-point Likert-type scale (ranging from 1 = never to 4 = often) how often each of the 20 statements reflected their experience in general. Total scores range from 20 to 80, where higher total scores indicate higher loneliness. The UCLA Loneliness Scale exhibited high internal consistency at its development (α = .96)[44] as well as in this study (α = 0.93 to 0.95 across time points). This scale has also been used in studies of gay men[45] and of HIV+ men.[46]

#### Fear of being sexually rejected

The Fear of Being Sexually Rejected Scale is an 8-item measure assessing the extent to which participants experience concern with being negatively evaluated or rejected by others if they express the desire to use a condom during sexual activity (i.e., “My sexual partner will think I am weird”). Participants indicated on a 5-point Likert-type scale (ranging from 1 = strongly disagree to 5 = strongly agree) the degree to which they agreed with each statement. Total scores range from 8 to 40, where higher total scores indicate greater concerns of sexual rejection. In this sample, Cronbach’s α coefficients for the Fear of Being Sexually Rejected Scale were high, 0.94, 0.94, and 0.93 at baseline, post-intervention, and 3-month follow-up, respectively.

#### Social cognitive theory constructs

The Social Cognitive Theory Constructs (SCTC) questionnaire was used to assess participants’ degree of self-efficacy to engage in sexual risk reduction behaviors.[47] Participants indicated the extent they agreed with each of the 5 statements on a 4-point Likert-type scale (ranging from 1 = strongly disagree to 4 = strongly agree). Total scores for self-efficacy range from 5 to 20, where higher total scores indicate higher self-efficacy. Cronbach’s α coefficients for the SCTC self-efficacy subscale were high, 0.80, 0.76, and 0.77 at baseline, post-intervention, and 3-month follow-up, respectively.

#### Sexual compulsivity

To assess the extent to which participants experienced “insistent, repetitive, intrusive, and unwanted urges” to engage in specific sexual acts, the 10-item Sexual Compulsivity Scale (SCS)[48] was administered. Participants indicated how characteristic each statement was of their experience on a 4-point Likert-type scale (ranging from 1 = not at all like me to 4 = very much like me). Total scores range from 10 to 40, where higher total scores indicate higher sexual compulsivity. The SCS demonstrates high internal consistency (α = .86) and moderate test-retest reliability among gay men (r = .64).[48] In this study, the SCS exhibited high internal consistency (α = 0.87–0.90 across all 3 timepoints).

#### Sexual sensation seeking

The Sexual Sensation Seeking Scale (SSSS)[49] consists of 11-items to assess the extent to which participants seek novel, thrilling, and adventurous sexual experiences and were susceptible to boredom and disinhibition during sexual situations. Participants indicated how characteristic each statement was of their experience on a 4-point Likert-type scale (ranging from 1 = not at all like me to 4 = very much like me). Total scores range from 11 to 44, where higher total scores indicate greater sexual sensation seeking. The SSSS demonstrates high internal consistency (α = .79) and moderate test-retest reliability among gay men (r = .69).[48] Cronbach’s α coefficients for the SSSS in this sample were 0.79, 0.76, and 0.75 at baseline, post-intervention, and 3-month follow-up, respectively.

#### Sexual behavior

Participants reported the total number of male sexual partners (ordinal: none, 1, 2–4, 5–9, 10+) as well as whether they engaged in anal sex with 1) a regular male partner(s), defined as a boyfriend, buddy, partner, or spouse (yes/no), 2) casual male partner(s) (yes/no), or vaginal or anal sex with 3) a regular female partner(s) (yes/no), or 4) casual female partner(s) (yes/no) over the past 3 months. In addition, participants indicated the HIV-status of their partner(s) (i.e., positive, negative, unknown status) and how often, over the prior three months, they engaged in receptive or insertive anal intercourse, with or without a condom, with their partner(s) (ordinal: never, once, 2 to 4 times, or 5 or more times).

#### Viral load

Participants self-reported their HIV viral load and were provided the option to indicate whether their viral load was detectable, undetectable, or unknown to the participant. Viral load was examined in the context of CAS, as CAS that occurred during a period of undetectable viral load confers significantly less HIV transmission risk to HIV-negative partners.

### Data Analysis

All analyses were conducted using SAS version 9.4 (SAS Institute, Inc., Cary, NC). Frequencies and univariate analyses (for age and years diagnosed with HIV) were calculated for socio-demographic variables. Additional frequencies related to specific sexual behaviors were also calculated. Univariate analyses were conducted for each psychosocial measure used in these analyses. Data are reported at each time period: baseline, post-intervention, and 3-month follow-up.

Since this analysis was conducted on identical data collected from participants at three different time points, a longitudinal approach to analysis was used that considers the high correlations between individual participants (using the REPEATED function). Repeated measures analyses were conducted using generalized estimating equations [50] in PROC GENMOD or linear mixed models [51] in PROC MIXED, depending on the type of outcome being studied (categorical or continuous, respectively). Separate data sets were created for each outcome, “stacking” the data for repeated measures analyses. Using the PROC GENMOD generalized estimating equation technique for a binary distribution, the time point was the predictor fit for each repeated measure, comparing post-intervention and follow-up measures to baseline values. For CAS as an outcome, we examined whether CAS was reduced over time both with and without controlling for whether CAS occurred when a participant had a detectable viral load. Using PROC MIXED for continuous outcomes, we used restricted maximum likelihood (REML) estimates for linear regression models. For both types of outcomes, an unstructured covariance structure was utilized so as to not assume a specific covariance structure across repeated measures. Analyses were limited to participants who completed the program. Because participants in this trial completed the trial in small groups, group-level clustering was considered in both PROC GENMOD and PROC MIXED procedures. For all analyses, statistical significance was determined at the α = 0.05 level.

## Results

### Sample Characteristics

Sample characteristics (n = 59) are summarized in Table 1. The majority of participants self-identified as White (63.8%). Our recruitment methods resulted in a similar distribution of ethnic categories as the MSM exposure category of new HIV diagnoses in Canada [52]. Regarding education, most participants indicated that they completed a high school or higher degree. Many participants (45.8%) reported currently receiving disability or pension payments and over half of participants (51.7%) had yearly incomes of less than CDN$20,000. The majority of participants (69.0%) self-reported having an undetectable viral load.

**Table 1:** Socio-Demographic and Clinical Characteristics of Gay Poz Sex (GPS) Pilot Program Completers and Non-Completers at Baseline.

	Completers (*n* = 59)	Non-Completers (*n =* 23)	
Variable	*n (%)*	*n (%)*	p-value
**Ethnicity**			
White	37 (63.8)	17 (77.3)	0.4172^a^
Black	9 (15.5)	2 (9.1)	
Latin American	7 (12.1)	1 (4.6)	
South Asian	2 (3.5)	1 (4.6)	
East/Southeast Asian	3 (5.2)	0 (0.0)	
Aboriginal	0 (0.0)	1 (4.6)	
**Highest education**			
Some high school	5 (8.5)	0 (0.0)	0.4367^a^
Completed high school	11 (18.6)	7 (31.8)	
Some secondary education	12 (20.3)	5 (22.7)	
Completed secondary education	23 (39.0)	9 (40.9)	
Completed graduate or professional school	8 (13.6)	1 (4.6)	
**Employment status**			0.5142^a^
Full-time	11 (18.6)	1 (4.8)	
Part-time	12 (20.3)	4 (19.1)	
Student	3 (5.1)	1 (4.8)	
Disability/Pension	27 (45.8)	11 (52.4)	
Unemployed	6 (10.2)	4 (19.1)	
**Annual income**			
Under $20 000	30 (51.7)	18 (81.8)	0.0968^a^
$20 000 - $39 999	13 (22.4)	1 (4.6)	
$40 000 - $59 999	9 (15.5)	1 (4.6)	
$60 000 - $99 999	4 (6.9)	2 (9.1)	
Over $100 000	2 (3.5)	0 (0.0)	
**Viral Load**			
Detectable	12 (20.7)	5 (21.7)	0.6086^a^
Undetectable	40 (69.0)	14 (60.9)	
Unknown	6 (10.3)	4 (17.4)	
	*M (SD)*	*M (SD)*	
**Age**	42.4 (9.0)	41.7 (8.3)	0.7968^b^
**Years since HIV diagnosis**	10.5 (7.5)	14.0 (8.1)	0.0781^b^

^a^Fisher’s Exact test

^b^ t-test comparison

### Sexual Behaviors

#### Individual participant behaviors

Frequencies of individual sexual behaviors are summarized in Table 2. Due to the nature of the program’s eligibility criteria, having had any “sex” (sexual activity beyond kissing, broadly-defined) over the course of the prior 3 months, was high. The majority of participants had had any type of “sex” over the prior three months with a casual partner at baseline (76.9%), post-intervention (67.3%), and 3-month follow-up (65.4%). Breaking this variable down by the casual partner(s)’ HIV-status(es) and specific behaviors, approximately one quarter of participants (23.1%) had insertive anal sex with a condom with an unknown HIV-status partner within the past 3 months at baseline, with a reduction (17.3%) at post-intervention, and a similar proportion at 3-month follow-up (25.0%). Similarly, 23.1% of participants had receptive anal sex with a condom over the prior 3 months with an unknown status casual partner at baseline, with 17.3% at post-intervention and 19.2% at follow-up. Insertive anal sex without a condom with an HIV-negative casual partner was low, with 11.5% reporting this behavior at baseline and post-intervention, and 7.7% at 3-month follow-up. A larger proportional decrease was seen in insertive anal sex without a condom with an unknown HIV-status casual partner, where 23.1% reported this activity at baseline, 9.6% at post-intervention, and 11.5% at 3-month follow-up. There were also changes seen in proportions of participants having had receptive anal sex (with any status partner) without a condom between baseline (59.6%) and 3-month follow-up (34.6%), with a slight reduction also seen at post-intervention (50.0%). There was also a large decrease in frequencies of participants having had receptive anal sex with an unknown HIV-status casual partner without a condom between baseline (32.7%), post-intervention (23.1%), and 3-month follow-up (13.5%).

**Table 2:** Condomless Anal Sex (CAS) in participants of the gay Poz Sex Pilot Program: Descriptive and Repeated Measures Modelling (Generalized Estimating Equations).

	Baseline (reference)		Post-Intervention (compared to Baseline)				Follow-up (compared to Baseline)			
Variable		n (%)		n (%)	Ο (95% CI)	p value		n (%)	OR (95% CI)	p value
CAS with casual HIV-negative partners	YES	9 (17.3)	YES	11 (21.2)	1.38 (0.64, 2.98)	0.4047	YES	9 (17.3)	1.06 (0.42, 2.69)	0.9001
	NO	43 (82.7)	NO	41 (78.9)			NO	43 (82.7)		
**CAS with casual HIV-positive partners**	YES	32 (61.5)	YES	24 (46.2)	**0.54 (0.29, 0.99)**	0.0456*	YES	22 (42.3)	**0.46 (0.25, 0.82)**	0.0090*
	NO	20 (38.5)	NO	28 (53.9)			NO	30 (57.7)		
**CAS with casual unknown HIV-status partners**	YES	22 (42.3)	YES	14 (26.9)	**0.55 (0.32, 0.98)**	0.0411*	YES	11 (21.1)	**0.37 (0.18, 0.76)**	0.0069*
	NO	30 (57.7)	NO	38 (73.1)			NO	41 (78.9)		
**CAS with casual partners (any status)**	YES	38 (73.1)	YES	30 (57.7)	**0.49 (0.26, 0.92)**	0.0259*	YES	26 (50.0)	**0.34 (0.17, 0.68)**	0.0022*
	NO	14 (26.9)	NO	22 (42.3)			NO	26 (50.0)		
CAS with HIV-negative partners (regular/casual)	YES	10 (19.2)	YES	11 (21.2)	1.38 (0.64, 2.98)	0.4047	YES	9 (17.3)	1.06 (0.42, 2.69)	0.9001
	NO	42 (80.8)	NO	41 (78.9)			NO	43 (82.7)		
**CAS with HIV-positive partners (regular/casual)**	YES	38 (73.1)	YES	28 (53.9)	**0.44 (0.25, 0.79)**	0.0057*	YES	27 (51.9)	**0.42 (0.24, 0.75)**	0.0030*
	NO	14 (26.9)	NO	24 (46.2)			NO	25 (48.1)		
**CAS with unknown HIV-status partners (regular/casual)**	YES	23 (44.2)	YES	15 (28.9)	**0.52 (0.29, 0.92)**	0.0253*	YES	11 (21.2)	**0.35 (0.17, 0.72)**	0.0043*
	NO	29 (55.8)	NO	37 (71.2)			NO	41 (78.9)		
**CAS with HIV-negative or unknown status partners (regular/casual)**	YES	28 (53.9)	YES	19 (36.5)	0.58 (0.32, 1.04)	0.0693	YES	15 (28.9)	**0.40 (0.20, 0.80)**	0.0103*
	NO	24 (46.2)	NO	33 (63.5)			NO	37 (71.2)		
**CAS with HIV-negative/unknown status partners (regular/casual), participant viral load unknown/detectable**	YES	26 (50.0)	YES	17 (32.7)	**0.53 (0.28, 0.99)**	0.0458*	YES	15 (28.9)	**0.46 (0.22, 0.96)**	0.0380*
	NO	26 (50.0)	NO	35 (67.3)			NO	37 (71.2)		
**CAS with all status partners, regular/casual**	**YES**	**44 (84.6)**	**YES**	**34 (65.4)**	**0.37 (0.19, 0.72)**	0.0033*	**YES**	**30 (57.7)**	**0.29 (0.14, 0.59)**	0.0006*
	**NO**	**8 (15.4)**	**NO**	**18 (34.6)**			**NO**	**22 (42.3)**		

*significant at α = 0.05

OR, odds ratios

CI, confidence interval

CAS, condomless anal sex

There were few changes in proportions of activities with a regular partner of any HIV status. Overall, over a third of participants reported having had any type of anal sex with a regular partner at baseline (34.6%), with more at post-intervention (36.4%) and fewer at 3-month follow-up (34.6%). The proportion of participants reporting insertive anal sex with a condom with a regular partner at baseline was 7.7%, 11.5% at post-intervention, and 9.6% at 3-month follow-up, respectively. Similar results were seen for receptive anal sex with a condom at baseline (7.7%), post-intervention (13.5%), and 3-month follow-up (13.5%). Higher proportions were seen with sexual activity without condoms, where 25.0%, 19.2%, and 19.2% reported insertive anal sex without a condom with a regular partner at baseline, post-intervention, and 3-month follow-up, respectively. Slightly larger proportions were found when examining receptive anal sex without a condom with a regular partner, with 28.9%, 30.8%, and 17.3% reporting this activity at baseline, post-intervention, and 3-month follow-up, respectively.

#### Condomless anal sex (CAS)

When specifically observing CAS (Table 2), the majority of participants reported CAS at baseline (84.6%) with fewer at post-intervention (65.4%), and 3-month follow-up (57.7%). The majority of participants reported any type of anal sex with a casual HIV+ partner without a condom at baseline (61.5%), with less than half reporting this behavior at post-intervention (46.2%) and 3-month follow-up (42.3%). Examining any CAS with casual HIV-negative partners, we see similar proportions across all time points (17.3%, 21.2%, and 17.3%); however, there is a large decrease in this behavior with casual partners of unknown HIV-status, with 42.3% reporting CAS at baseline, 26.9% at post-intervention, and 21.1% at 3-month follow-up.

When specifically examining CAS with a regular HIV+ partner, a reduction from 32.8% at baseline, 28.9% at post-intervention, and 25.0% at 3-month follow-up was observed. CAS with a regular HIV-negative partner was low at baseline (1.9%) and post-intervention (1.9%), and reduced to none at 3-month follow-up.

### Repeated Measures Analysis

#### Generalized estimating equations (GEE) and sexual behaviors

Individual changes in participants’ behaviors are summarized in Table 2. We used a composite variable based on the additive risk of HIV transmission during CAS. Namely, sexual activity was dichotomized into high or low risk of HIV transmission based on 2 dimensions: (1) participant’s own detectable or unknown versus undetectable viral load and (2) HIV-negative or unknown serostatus versus HIV+ sex partner. The proportion of participants who reported engaging in the higher category of risk in both dimensions (detectable/unknown viral load and HIV-negative/unknown serostatus partner) during CAS with a casual or regular partner reduced from baseline to post-intervention to 3-month follow-up from 50.0% to 32.7% to 28.9%, respectively.

Overall, we noticed several statistically significant reductions in individual behaviors. Examining frequencies of CAS with an unknown HIV-status partner, we observed a statistically significant reduction at follow-up compared to baseline (p = 0.0148). Similarly, we noticed a reduction in receptive CAS with both HIV+ (p = 0.0176) and unknown HIV status (p = 0.0121) casual partners at 3-month follow-up compared to baseline. No additional changes were seen over time and no statistically significant reductions in individual sexual activities were observed with regular partners.

Examining changes in the composite measure to create an outcome of CAS with HIV-negative or unknown status casual or regular partners when the participant had a detectable or unknown viral load, there was an overall reduction at post-intervention (p = 0.0458) and 3-month follow-up (p = 0.0380).

#### Linear regression and psychosocial measures

Table 3 summarizes results of repeated linear regression models examining psychosocial measures over time. On average, there were no statistically significant reductions in depression scores at post-intervention (p = 0.1886) or 3-month follow-up (p = 0.1110). Scores on the UCLA Loneliness Scale were significantly lower at both post-intervention (p = 0.0093) and 3-month follow-up compared to baseline (p = 0.0046). Similar reductions were seen with fear of being sexually rejected (p-value [post-intervention] = 0.0185; p-value [3-month follow-up] = 0.0014) and in the model examining sexual compulsivity (p-value [post-intervention] = 0.0014; p-value [3-month follow-up] = 0.0001). We found an increase in self-efficacy related to condom use, with statistically significant increases at both post-intervention (p = 0.0001) and 3-month follow-up (p<0.0001), compared to baseline. A statistically significant reduction in sexual sensation seeking was seen at post-intervention (p = 0.0422), however, this measure did not remain statistically different at 3-month follow-up (p = 0.1746).

**Table 3:** Psychosocial Measures of Participants in The Gay Poz Sex Pilot Program: Descriptive and Repeated Measures Modelling Results (Linear Regression).

	Baseline (reference)	Post-intervention (compared to Baseline)			Follow-up (compared to Baseline)		
Measure	Mean (SD; n)	Mean (SD; n)	β (95% CI)	p value	Mean (SD; n)	β (95% CI)	p value
CES-D (Depression)	16.0 (10.2; 47)	13.5 (11.1; 45)	-2.11 (-5.26, 1.05)	0.1886	13.3 (10.6; 48)	-2.54 (-5.67, 0.59)	0.1110
UCLA (Loneliness)	46.7 (10.3; 50)	42.7 (11.0; 47)	**-4.08 (-7.14, -1.02)**	0.0093*	42.4 (12.4; 50)	**-4.10 (-6.92, -1.29)**	0.0046*
FBSR (Fear of being sexually rejected)	21.2 (8.1; 51)	18.4 (7.8; 51)	**-2.24 (-4.10, -0.38)**	0.0185*	17.6 (6.7; 50)	**-3.14 (-5.04, -1.24)**	0.0014*
SCTC (Social cognitive theory constructs)–self efficacy)	2.7 (0.7; 50)	3.1 (0.6; 52)	**0.35 (0.17, 0.53)**	0.0001*	3.1 (0.6; 50)	**0.39 (0.23, 0.54)**	<0.0001*
SCS (Sexual compulsivity scale)	25.1 (8.8; 49)	21.3 (8.4; 49)	**-3.34 (-5.37, -1.31)**	0.0014*	20.6 (8.2; 51)	**-4.27 (-6.43, -2.10)**	0.0001*
SSSS (Sexual sensation seeking scale)	31.6 (5.8; 51)	29.7 (5.7; 50)	**-1.49 (-2.93, -0.05)**	0.0422*	30.7 (5.3; 48)	-1.04 (-2.56, 0.47)	0.1746

*significant at α = 0.05

SD, standard deviation; CI, confidence interval; CES-D, Center for Epidemiologic Studies–Depression Scale; UCLA, University of California, Los Angeles–Loneliness Scale; SCTC, Social Cognitive Theory Constructs–Self-Efficacy; SCS, Sexual Compulsivity Scale; SSSS, Sexual Sensation Seeking Scale

## Discussion

Findings from this pilot trial provide strong preliminary evidence for the acceptability and efficacy of GPS health counseling for HIV+ GBM in reducing HIV sexual transmission risk behaviors. CAS with HIV-negative and unknown HIV-status partners reduced from 53.9% at baseline to 28.9% at 3-month follow-up. Findings were robust even when controlling for whether or not the participant had a detectable or unknown viral load. Our secondary hypothesis that the intervention would be associated with reduced CAS with HIV+ partners was also supported, with a reduction from 73.1% at baseline to 48.1% at 3-month follow-up. The statistically significant reductions in CAS are promising, especially given that the trial was designed primarily as a Phase I trial and not to detect statistically significant reductions in CAS.

Significant reductions were also found in loneliness, sexual compulsivity, and fear of being sexually rejected for insisting on condom use. Few treatments have been designed for people living with HIV that simultaneously result in reductions in CAS and common mental health problems in this population, such as loneliness[45,53] and sexual compulsivity.[54] Furthermore, these findings are consistent with the syndemic model of co-occurrence of health risk behaviors and mental health problems among marginalized populations such as GBM.[53, 55–57] To our knowledge, our study is the first to reduce both sexual health risk behaviors and mental health difficulties for HIV+ GBM concurrently via health counseling.

The reduction in sexual compulsivity is particularly noteworthy, as it has been significantly linked to a number of sexual risk behaviors, including CAS,[38,58] as well as clinical outcomes, such as sexually transmitted infections.[59] Although the causal mechanism between sexual compulsivity and increased sexual risk is not yet clearly known, it has been suggested that sexual risk behaviors occur through acute deficits in rational decision-making due to prolonged periods of sexual arousal[60] or a reduction in inhibitions due to the increased use of “club drugs.”[38]

Given the focus of GPS on having sex in accordance with one’s personal sexual health goals, we suspect the reduction in sexual compulsivity may be due to participants engaging in more desired sexual activities, which may have subsequently decreased their sense of a lack of sexual control. It may be that the overall reduction found in CAS with HIV+ and HIV-negative partners is, at least partly, attributable to reductions in sexual compulsivity; unfortunately, this study’s sample size does not permit an examination of this possibility. Notably, the intervention did not reduce sexual sensation seeking, which refers to the propensity to seek out novel and uninhibited sexual stimulation. This finding is consistent with the intervention’s goal to decrease risks of transmitting HIV while simultaneously respecting each participant’s sexual interests (i.e., helping men to “get the sex you want” without restricting sexual activity). As a result, this intervention also adequately responds to recent calls to better integrate mental health into patient care for people living with HIV in order to address CAS in the context of syndemic problems.[61,62]

Although there was a marginal decrease in mean scores of depressive symptomatology from 16 (SD = 10.2) at baseline to 13.5 (SD = 11.1) at post-intervention and 13.3 (SD = 10.6) at 3-month follow-up, the reductions were not statistically significant. It is unsurprising that the intervention did not significantly reduce depressive symptomatology, since motivational interviewing is not a treatment typically used to treat depression. However, the reductions may have been meaningful from a clinical perspective, since the mean baseline score of 16 is the recommended cutoff score on the CES-D to indicate that further assessment for depression is warranted,[41] and the mean scores were below this cutoff after the intervention.

Although the study provides preliminary data about the potential for GPS to reduce CAS and psychological distress among HIV+ GBM in the real-life, ecologically-valid setting of an AIDS service organization, the study design, as a one-armed trial, needs to be followed-up with a rigorous test of the efficacy of GPS using a randomized controlled design. The name of the intervention, Gay Poz Sex, may have reduced the chances that non-gay identified MSM felt welcome to participate in the study, although it is likely that most men who wish to participate in a sexual health promotion program for MSM would be gay-identified. The study is also limited in its ability to test the underlying IMB theoretical model, including the mediating effects of GPS on HIV-related information, motivation, and behavioral skills. Although reducing loneliness, sexual compulsivity, and fear of being sexually rejected were part of the study’s goals, as previously mentioned, the study’s smaller sample size precludes assessing whether reductions in CAS at follow-up were due to reductions in these study variables. The dropout of 23 men from the completer sample limits our full understanding of the potential effects of the program.

With increased use of HIV biomedical treatment as prevention including for HIV-negative persons,[29,63,64] data are also needed showing how sexual behavior changes among GPS participants would be affected by their perceptions of having an undetectable viral load or by use of pre-exposure prophylaxis among their HIV-negative partners. The self-report methods used in the present study could be affected by social desirability response bias, with a potential bias in a favorable direction over time. An examination of medical outcomes resulting from GPS, such as sexually transmitted infections and objectively measured viral load would strengthen the evidence base for GPS.

Despite these limitations, the GPS intervention innovatively applies an empirically supported model for sexual risk reduction (i.e., IMB model) in order to simultaneously reduce CAS and mental health problems that are reliable risk factors for CAS. GPS and other counselling programs continue to be important as companion methods to biomedical HIV prevention methods, such as treatment as prevention and pre-exposure prophylaxis, due to an increasing STI epidemic among MSM and the lack of evidence of a decrease in HIV incidence among MSM [65] despite increases in the prevalence of suppressed viral load among HIV-positive MSM. In addition, the intervention, which was administered by paraprofessional HIV+ peer counselors, offers an implementation model that is more easily generalizable to real-life settings, as it does not necessitate treatment by a mental health professional. Consistent with other treatments developed with an eye toward cost-effective use of clinical and therapy resources,[66] our use of paraprofessional peer facilitators means that the intervention can be delivered by people from a range of educational levels and disciplinary backgrounds. This intervention therefore may also be cost-effective to administer in both clinical and community settings without using the resources of mental health clinicians. The study findings suggest that GPS may offer an efficient way of concurrently reducing CAS and mental health problems for HIV+ GBM.

## Supporting Information

S1 TREND Checklist (PDF)

S1 Approval (ZIP)

S1 Dataset (XLSX)

S1 Protocol (DOC)
