# Supplementary material for: A Pilot Trial of a Sexual Health Counseling Intervention for HIV-Positive Gay and Bisexual Men Who Report Anal Sex without Condoms
Source: PLoS One. 2016 Apr 7;11(4):e0152762. doi: 10.1371/journal.pone.0152762 (PMC4824469; doi:10.1371/journal.pone.0152762)
Supplement: S1 Approval — (ZIP) [file pone.0152762.s002.zip › REB approval/Approval email October 09, 2007 11_29_36 PM.pdf]

# Email sent to applicant on October 09, 2007 11:29:36 PM

From: rebchair@ryerson.ca
To: trevor.hart@ryerson.ca
Cc: rebchair@ryerson.ca
Subject: REB 2007-176 Status
Date: October 09, 2007 11:29:36 PM

Dear Trevor Hart,

Dr. Trevor Hart
Psychology

REB 2007-176
Development of an HIV Prevention and Sexual Health Intervention for Positive Men

The Research Ethics Board has completed its review of your submission. Your research project is now approved. The approval letter is attached in Adobe Acrobat (PDF) format.

Congratulations and best of luck with the project.

Please quote your REB file number (REB 2007-176) on future correspondence.

If you have any questions regarding your submission or the review process, please do not hesitate to get in touch with the Research Ethics Board (contact information below).

No research involving humans shall begin without the prior approval of the Research Ethics Board.

Record respecting or associated with a research ethics application submitted to Ryerson University.

Yours sincerely,

Alex Karabanow on behalf of
Nancy Walton, Ph.D.
Chair, Research Ethics Board

Alexander Karabanow
Office of the Vice President, Research and Innovation
Ryerson University, 350 Victoria Street, Room YDI 1154
Toronto, Ontario, Canada M5B 2K3
Phone: (416) 979-5000 Ext. 7112, Fax: (416) 979-5336
Email: alex.karabanow@ryerson.ca Web: http://www.ryerson.ca/research

---

Print Email | Close Window
