# Supplementary material for: A Pilot Trial of a Sexual Health Counseling Intervention for HIV-Positive Gay and Bisexual Men Who Report Anal Sex without Condoms
Source: PLoS One. 2016 Apr 7;11(4):e0152762. doi: 10.1371/journal.pone.0152762 (PMC4824469; doi:10.1371/journal.pone.0152762)
Supplement: S1 Approval — (ZIP) [file pone.0152762.s002.zip › REB approval/Submission September 2007.pdf]

APPLICATION CHECKLIST

Protocol Submitted By: trevor.hart

Submission Status: Submitted for review

REB: 2007-176

Comments to Chair

Title of Research Proposal
Development of an HIV Prevention and Sexual Health Intervention for Positive Men

Principal Investigator

Type:	Other: University of Windsor Faculty
First Name:	Barry
Last Name:	Adam
Institution:	University of Windsor
Academic Title:	
Department/Office:	Sociology and Antropology
Email:	adam@uwindsor.ca
Telephone Number:	519-253-3000 x3497

Co-Investigator(s)

First Name:	David
Last Name:	Hoe
Institution:	
Academic Title:	
Department/Office:	
Email Address:	davidhoe@magma.com
Telephone Number:	

First Name:	Eleanor
Last Name:	Maticka-Tyndale
Institution:	University of Windsor
Academic Title:	
Department/Office:	Sociology and Anthropology
Email Address:	maticka@uwindsor.ca
Telephone Number:	416-586-4800

First Name:	Herbert
Last Name:	Co
Institution:	Toronto Public Health
Academic Title:	
Department/Office:	
Email Address:	coherb2000@yahoo.com
Telephone Number:	

First Name:	James
Last Name:	Murray
Institution:	Ministry of Health and Long-Term Care
Academic Title:	
Department/Office:	AIDS Bureau
Email Address:	james.murray@moh.gov.on.ca
Telephone Number:	800-268-6066

First Name:	John
Last Name:	Maxwell
Institution:	AIDS Committee of Toronto
Academic Title:	
Department/Office:	
Email Address:	jmaxwell@actoronto.org
Telephone Number:	416-340-8484

First Name:	Robert
Last Name:	Leahy
Institution:	
Academic Title:	
Department/Office:	
Email Address:	baxter@accel.net
Telephone Number:	

First Name:	Robert
Last Name:	Mackay
Institution:	
Academic Title:	
Department/Office:	
Email Address:	rajm@tbaytel.net

Telephone Number:

First Name: Trevor
Last Name: Hart
Institution: Ryerson University
Academic Title:
Department/Office: Psychology
Email Address: trevor.hart@ryerson.ca
Telephone Number: 416-979-5000 x6192

Student Researcher(s)

No student researchers were specified in this protocol.

Access to Protocol

No investigators, other than trevor.hart, have been granted access to this protocol.

Has this protocol been approved or is currently being reviewed by other Research Ethics Boards? Yes

Institution: University of Windsor
Status: Approved
Attached Materials:
- University of Windsor application (attachment)

Projected Dates of Data Collection and Analyses

Begin Recruitment Date: November 1, 2007
End Analysis Date: October 1, 2010

Funding Source

Funding application pending

Sponsor(s)

Name: CIHR
Reference Number:
Funding Period Begin: November 01, 2007
Funding Period End: October 31, 2010

If the study is funded, will the Principal Investigator require the approval of the sponsor(s) before the publication of the findings? No

Does the Principal Investigator(s) or Co-Investigator(s) have a financial interest or personal relationship with member(s) of the funding sponsor(s)? No

Use of Existing Data

Will this study involve the use of existing data, documents, records, pathological specimens, or diagnostic specimens? No

Participants to be Recruited

No participants have been selected.

Data Will Include

No data includes have been selected.

Are codes used to link data to participants? Yes

Is compensation offered? Yes

Please state the type and amount of compensation or reimbursement of expenses; whether a per diem for food, travel or time, or an honorarium, etc.:

Type: Honorarium
Amount: $25 for each session

Why do you think that amount and form of compensation is appropriate?

All participants will receive $25 for each of 3 quantitative assessments, 10 will receive $25 for each of 3 qualitative interviews. $125 has been budgeted per worship to be used for raffled gift cards to sustain attendance in the intervention. Participants will also be provided subway tokens to travel to workshops.

Number of Participants

No participants were specified in this protocol.

Method of Recruiting

Will potential participants be involved in:

- **An intervention or manipulation?** No
- **Deception?** No

Potential Risk Exposures

Psychological
Social

If you think your protocol is minimal in risk to your participants, state why you think it is minimal risk?

It is possible, though unlikely, that the protocol may raise issues that may be troublesome to some of the participants. A fundamental objective of the project is the creation of workshops designed to work through issues of sexual health and HIV risk [illegible]

management. Another risk for participation in the study is related to breaks in confidentiality. Several steps will be taken so that data will remain confidential both during and after the investigation.

Are there any issues of cultural diversity with respect to privacy that you are aware of in the questions you're asking and the type of participants you are recruiting?

Instruments

Data will be recorded by:

Method of data collection:

Findings used for:

Method of obtaining consent:

- Written consent form

Method of obtaining consent/assent from children or participants unable to sign legally valid consent:

- No written assent, under the age of 7 (include information on how the researcher will determine consent and monitor the participant's willingness to continue in the study).

  More Information:

STUDY ABSTRACT

Please provide a one paragraph summary of the protocol that includes a brief description of the methods, potential benefits, potential risks, and risk management procedures.

Participating in this program involves the following: filling out a questionnaire on the participants views, feelings, and practices concerning meeting and having sex with men, coming to 6-8 evenings on issues that come up in living with HIV and in meeting and having sex with men, letting us know how valuable (or not) each of these evenings are through an evaluation form. We then want to contact the participant 3 months after the program ends to see if views, feelings, and practices have changed over time. All participants will receive $25 for each of 3 quantitative assessments, 10 will receive $25 for each of 3 qualitative interviews. $125 has been budgeted per worship to be use for raffled gift cards to sustain attendance in the intervention. Participants will also be provided subway tokens to travel to workshops. One immediate benefit is that subjects will be introduced to strategies of HIV prevention and sexual health that may work better for them than previous strategies that they have been exposed to. Also, subjects may feel some amount of satisfaction at participating in a study to help decrease the HIV infection in the gay community. Some participants may appreciate the study's focus on mental health and its in-depth focus on what may actually be happening in sexual situations that may inhibit or promote healthy sexual behaviour. The project also has a well-developed referral system built into it to assist men with depression, substance abuse, and related issues. Completion of the assessments are free of risks for physical harm. It is possible, though unlikely, that the assessment may raise issues that may be troublesome to some of the participants. A fundamental objective of the project is the creation of workshops designed to work through issues of sexual health and HIV risk management. Another risk for participation in the study is related to breaks in confidentiality. Several steps will be taken so that data will remain confidential both during and after the investigation. Program facilitators will include a health professional, and the project will be housed at the AIDS Committee of Toronto which has HIV-related counselling and information resources, should the need for referrals arise. Research staff will be supervised by a licensed clinical psychologist (Dr. Hart) who is experienced in working with seropositive men. Since the intervention uses a workshop format, participants will be able to become acquainted with each other. Participants will be advised that they should respect each other's privacy but to keep in mind that what is said inside workshops cannot be guaranteed to be kept confidential by other participants. This advice is given in accord with the policy used by the AIDS Committee of Toronto in support groups with HIV-positive people. Threats to confidentiality will be minimized by using code numbers, not names for assessments. Because sexual behaviour data are being collected by an ACASI system, these data will be automatically stored on a computer, and there will be no paper copies of data with code numbers. At the end of data collection sessions, a staff member will download all data records from the laptop computer onto USB key drives. The USB key drives will be transported immediately to the data manager's computer for storage and compilation with previously collected data as a master data file. All data will be stored in a password-protected master file and be backed up in a password-protected file. All confidential material including code numbers and data will be kept in a locked office in locked file cabinets with limited access.
