# Supplementary material for: A Pilot Trial of a Sexual Health Counseling Intervention for HIV-Positive Gay and Bisexual Men Who Report Anal Sex without Condoms
Source: PLoS One. 2016 Apr 7;11(4):e0152762. doi: 10.1371/journal.pone.0152762 (PMC4824469; doi:10.1371/journal.pone.0152762)
Supplement: S1 Protocol — (DOC) [file pone.0152762.s004.doc]

**1.0 OUTLINE OF THE PRESENT STUDY**

Among men who have sex with men (MSM) in Ontario, from 2001 to 2006, HIV diagnoses increased 26%^1^. In 2006, there were 522 new cases of HIV among MSM, and MSM accounted for 65.2% of HIV of new cases in Ontario (including 2.8% who are MSM and injection drug users). An estimated 19.6% of Toronto MSM are HIV-positive (HIV+)^2^. Behavioural trends in the same time period show that seroconversions are not randomly distributed across the population of gay and bisexual men. Unprotected anal intercourse (UAI) appears to occur more frequently among HIV+ versus HIV-negative MSM. For example, in a study of HIV+ MSM recruited in both 2005 and 2007, 37.6% of MSM had engaged in UAI with partners of unknown or serodiscordant HIV status, as opposed to 13.3% of HIV-negative MSM^3^. Among both HIV+ and HIV-negative men, half of all the unprotected sex reported in the previous six months occurred among those who identified themselves as participants in the “bareback scene” or “bareback websites” (i.e. venues facilitating intentional UAI)^4,5^.

Despite continued UAI among HIV+ persons, the vast majority of prevention efforts have focused on HIV-negative populations. Further, few intervention studies to our knowledge have specifically examined the effects of an intervention intended for HIV+ MSM. However, given the potentially greater impact on the HIV epidemic of decreasing sexual risk behaviour of HIV+ persons, it is critical that these interventions be developed for HIV+ MSM, and that these interventions acknowledge the recent effect of barebacking-related beliefs among certain groups of MSM^6^.

The primary objective of the present study is to determine the effect size of a theoretically-guided HIV prevention program for HIV+ MSM to reduce HIV transmission risk behaviour using a community-based research framework. An effective HIV prevention intervention for HIV+ MSM has the potential to make a significant difference in epidemiological trends, given the high HIV prevalence rate among MSM in Canada and increasing HIV incidence. An effective intervention also may have wider health benefits for HIV+ men by reducing exposure to co-infection with hepatitis, Lymphogranuloma venereum, syphilis, and other sexually transmitted infections (STIs), and with alternative strains of HIV^7,8^.

**2.0 BACKGROUND AND RATIONALE**

**2.1 UAI with HIV-negative or unknown serostatus partners among HIV+ MSM**

Several studies suggest that many MSM operate under mistaken assumptions about their sexual partners’ HIV status. Many HIV+ men read the willingness of their partners to engage in UAI as itself evidence that partners are already HIV+^9-18^. HIV-negative men, on the other hand, may assume the opposite: that partners willing to engage in UAI “must be” negative^19,20^. However, these assumptions may frequently be mistaken, as 64% of partners of HIV+ persons who were perceived by participants as HIV+ were actually HIV-negative^11^.

Another important factor that may account for the increasing HIV prevalence among MSM in Ontario is the rise of barebacking, often defined as intentional UAI^4,22^. The development of sub-groups of men who bareback among HIV+ men may account for a cascade of recent evidence showing elevated and rising rates of UAI among HIV+ men with partners of unknown or negative HIV status^23-37^. The Ontario Men’s Survey^38^ found that 30% of HIV+ men reported having UAI with a partner of unknown serostatus during the previous year, and 20% with a partner they assumed to be HIV-negative. It is critical to note that while most (but not all) barebackers are HIV+, most HIV+ men are not barebackers^39^. Many practise safe sex and many more employ risk reduction techniques if a condom is not used. However, there is still an unmet need for empirically tested interventions to reduce sexual risk behaviour among HIV+ MSM, including those who engage in barebacking (intentional UAI) with non-HIV+ partners or who otherwise engage in UAI with non-HIV+ partners without a previous intention of engaging in UAI.

**2.2 Interventions to reduce sexual risk behaviour among HIV+ persons and MSM**

The most recent meta-analyses suggest that behavioural interventions are efficacious at reducing self-reported UAI among MSM (RR = 0.80, 95% CI 0.72-0.89)^40^. Among small group and individual-level interventions, significant effects were observed for interventions that addressed information, risk perception, self-efficacy, personal skills (e.g., self-reinforcement for behaviour change) and interpersonal skills related to sex. Effects found in multivariate analyses were observed for interventions that addressed perceptions of risk and losses, such as unprotected sex can harm you, versus those that addressed only gains from safer sex. Interventions were not differentially effective whether an intervention was delivered by peers versus others, or individual versus small-group based. Given that interventions outside the United States demonstrated a nonsignificant reduction in UAI^41^, and especially given the lack of efficacious interventions designed for Canadian populations of people living with HIV, there is a specific need for research investigating the effects of behavioural interventions in Canada.

Other reviews have found^42^ greater efficacy with more active interventions (i.e., participants role-play problem situations, or practice applying condoms to a model) rather than passive interventions where participants just receive information. An efficacious intervention among HIV+ persons^43^ used scenes drawn from films, plus active role playing, in a program delivered to primarily African American HIV+ men and women, both gay and straight, in: (1) developing skills to cope with HIV-related stressors and sexual risk producing situations, (2) enhancing decision-making skills for self-disclosing HIV serostatus to sexual partners, and (3) facilitating the development and maintenance of safer sexual practices. Other interventions report variations on this model focussing on improving self-efficacy in negotiating safer sex and/or disclosure skills ^44-49^.

There have been far fewer behavioural interventions specifically focusing on reducing HIV transmission risk behaviour among HIV+ MSM. The relative lack of behavioural interventions for HIV+ MSM is problematic because behavioural interventions including samples with more than 35% HIV+ MSM were not efficacious in a recent meta-analysis^41^. Another^40^ meta-analysis of the positive prevention literature reported that interventions were efficacious to the extent that they did not focus on HIV+ MSM. In one of the largest and most intensive interventions developed to date, the Seropositive Urban Men’s Intervention Trial (SUMIT) found that participants had less receptive UAI after the 6-week course, but the effect faded 3 months later^50^. The SUMIT investigators speculated that SUMIT’s lack of efficacy may have been due to the influence of some participants in small group discussions who “disclosed that they regularly had unprotected sex, that they believed that their partners should be responsible for their own health, and that they were not convinced that they could transmit HIV during receptive anal or insertive oral sex”. This is particularly noteworthy as these views are recognizable as central tenets of the bareback sub-group of MSM.

In summary, from previous intervention research and our own interviews with Toronto MSM having UAI most or all of the time^6^, it appears that it is necessary to engage the emerging culture of risky sex among HIV+ MSM if the incidence of UAI, especially with partners of negative or unknown serostatus, is to be reduced. The present study will address this challenge in Canada by engaging HIV+ MSM in a community setting and employing an empirically supported model, the Information-Motivation-Behavioral Skills^51,52^ focusing on information, motivation, and behaviour skills as a means to prevent HIV transmission risk behaviour. The IMB model will be delivered by motivational enhancement counseling (also known as “motivational interviewing”), a counseling technique that that expresses empathy for the participants, highlights differences between current behaviour and current health promotion goals, avoids argumentation with clients, and supports a participant’s self-confidence in his ability to engage in risk reduction behaviours^53-56^.

**2.3 Guiding Model for the Intervention: The IMB Model**

**2.3.1 Elicitation research using the IMB model**. The IMB model developed by Fisher et al. (2002) will serve as the theoretical model guiding the present study’s HIV prevention intervention^52^. This behavioural model, supported by PHAC’s Canadian Guidelines for Sexual Health Education^57^, is strongly empirically supported for a variety of populations regarding HIV risk prediction and in the development of efficacious HIV prevention interventions^23,58-62^. The IMB model specifies that HIV prevention information and motivation are relatively independent constructs which increase HIV preventive behaviour directly and by increasing behavioural skills (e.g. effective condom negotiation) that in turn increase HIV preventive behaviour. The IMB model specifies that elicitation research must first be conducted before designing HIV prevention interventions. Elicitation research refers to research that identifies relevant information, motivation, and behavioural skills of the target population, and typically includes focus groups, interviews, and surveys. This research has already been conducted by the study team on the information, motivation, and behavioural skills of HIV+ MSM in Ontario^4,14,15^ and is reviewed below:

**2.3.2 Information.** This is a population that is largely well-informed about the basics of HIV transmission and pathogenesi**s**^59^ and typically already in treatment. Based on our own research^4^, as well as that of others, HIV+ men need advanced information that could help them in their decision making processes regarding HIV risk, including: 1) the relationship between viral load and transmission^59^, 2) solutions to erectile problems^10,63,64^, 3) unprotected sex and acquisition of alternative strains of HIV, syphilis, LGV, hepatitis C, etc, 4) the risk of top or bottom roles in sex and the risk of sex with another HIV+ person^4^, 5) risks of presuming or intuiting a potential partner’s sero-status, and 6) the current legal context in Canada in which unprotected sex without disclosure may be considered a crime^65^.

**2.3.3 Motivation**. For this set of men who have unprotected sex much of the time, motivation is a critically important area to address. Men with bareback practices demonstrate inconsistent motivations regarding engaging in unprotected anal intercourse. For example, although they fairly consistently engage in UAI and are aware of risk of HIV transmission, few, if any, wish transmission to occur and most, if not all, articulate their desire to actively avoid transmission. Many also express a desire to give back to the gay community and to protect younger or uninformed men^14^.

**2.3.4 Behavioural skills.** While the central focus for risk reduction will be on condom use in anal intercourse, research documents the complex array of behaviours and skills required for consistent condom use^47-49, 58^. Based on the extant literature, it is anticipated that this intervention will address: (a) HIV-status disclosure skills; (b) negotiation of safer sex practices; (c) exploration of nonpenetrative sex alternatives; and (d) experimentation with a variety of condoms to enhance the condom-wearing experience, in a context of expanding ideas of what may be desirable and how to have sexy, satisfying, and intimate relations. Behavioural skills are typically delivered using both role play of behavioural skills and identification of high-risk situations (e.g. substance use, depression). Behavioural skills approaches can be integrated into motivational approaches to create efficacious interventions, and have been used in previous studies with heterosexual populations^58^.

**2.4 Intervention Implementation**

**2.4.1 The Intervention Implementation Model: Motivational Enhancement Counselling**

Per the IMB model, the study intervention will specifically address HIV prevention information, motivation, and behavioural skills. The intervention implementation model, will build directly from the IMB theoretical model using motivational enhancement counselling^53,55,58,59^. Motivational enhancement counselling is designed to induce rapid, internally motivated change by using the participants’ own change resources^58,60^. The specific steps include: 1) eliciting from the participant his ideas about how a behaviour can be changed, 2) enhancing the participant’s confidence in being able to make the desired change, while 3) constantly attempting to help the participant locate and express his own reasons for wanting to change^53^. Motivational enhancement counselling also tailors the intervention toward a participant’s stage of readiness to make change^54,66^. Considering the recurrence of social isolation as a theme in the narratives of risk-taking men^38,53,68-70^, the intervention will also be designed as a small group counselling session to help foster social interaction among participants. A major difference between the small group intervention for HIV+ MSM described by Wolitski et al (2004) and the small group intervention in the current study is the intervention implementation model. The unsuccessful SUMIT intervention used social cognitive theory, which posits that people make change when given sufficient information and behavioural skills^68,71,72^. In contrast, the intervention in the present study does not assume that information and behavioural skills are sufficient to make change, and instead relies also on helping participants to find their own internal motivations to engage in behavioural change. Motivational enhancement counselling is also a goal-directed counselling style that has been shown to be effective in reducing sexual transmission risk behaviour for primarily HIV-negative samples of MSM and high-risk heterosexuals^60,62^.

Informational component. In this component of the intervention, delivered primarily in Sessions 1 and 2, facilitators will review information about transmissibility of HIV and its association with HIV viral load and medical factors, discussion of other STIs and their effects on HIV disease, and strategies used by MSM to promote sexual health, and psychosocial problems that decrease sexual health. This component will use interactive activities to dispute myths about HIV (e.g., “Unprotected sex is not risky if you are already HIV+”) and to discuss risk which behaviours are of greater or lesser risk for HIV transmission.

Motivational component. Key to this approach is identification of areas of inconsistent or conflicting motivations. Working with participants’ own sometimes inconsistent beliefs, for example, that partners are (solely) responsible for HIV risk, but at the same time, expressing altruistic values of taking care of other gay men and contributing to gay community, offers significant starting points for carrying forward the techniques of motivational enhancement counselling. This component will be delivered primarily in sessions 2-6.

Behavioural skills component. In sessions 4-6, the facilitators will help participants manage cues that may serve as triggers for risky sexual behaviour, and to identify strategies to reduce their risk of contracting STIs and/or transmitting HIV. This component will include role-play exercises of risk-reduction skills, and will use the knowledge of the group to help individual group members to begin and maintain behavioural change.

It should be noted that although there are three different components of the intervention, the components are interlaced throughout the intervention, albeit at different amounts. For example, per motivational enhancement counselling, information provision is more appropriate at earlier stages of readiness for change^53,54^, and behavioural skills are more appropriate once intrinsic motivation is increased to a level in which the participant is ready to make behavioural changes.

**2.4.2 Structure of the Intervention**

The intervention will take the form of a small counselling group, since this is one of the most common intervention forms for similar populations. Recent studies have found positive effects on cardiovascular health behaviours (e.g., exercise, diet) using motivational enhancement counselling in a small group-forma**t^73^.** Participants will be organized into 7 groups of approximately 6 participants, each of which will be led by 2 facilitators who are HIV+ MSM. Months 1-4 will be spent completing the final version of the intervention manual, based upon data that will be received through focus groups with HIV+ MSM and with prevention and care providers for HIV+ MSM. These data come from the preliminary one-year project received by our team and will be completely analyzed by March 2009 and ready for incorporation into the manual. Per the IMB theoretical model and the motivational enhancement counselling treatment model, an outline of this manual has already been created and is found in Appendix 1.

**3.0 PRINCIPAL AIMS AND HYPOTHESIS OF THE PRESENT STUDY**

The current study will investigate an HIV prevention intervention for HIV+ MSM. This Phase II trial will determine the effect size of the intervention, while incorporating one aspect of a Phase I trial by examining the tolerability of the intervention among HIV+ MSM. The present study is intended to be a rigorous evaluation of the HIV prevention intervention, with the ultimate aim of evaluating the efficacy of the intervention using a Phase III randomized control trial (RCT), per standard procedures for evaluating health interventions^74^**.**

The study hypothesis is that the study intervention will result in reduced prevalence of unprotected anal intercourse (UAI) with partners who are HIV-negative or unknown serostatus.

**4.0 RESEARCH DESIGN**

**4.1 The Research Team**

The research team includes partners from universities, AIDS service organizations (ASOs), government, and the Poz Prevention Working Group (PPWG). The PPWG, the majority of its members who are themselves HIV+ MSM, has been working for more than a year on effective and sustainable HIV prevention for HIV+ MSM with the assistance of the AIDS Bureau of the Ontario Ministry of Health and Long Term Care. The partners bring expertise to the table in research, knowledge translation, intervention delivery, policy formulation and implementation, program evaluation, and multisectoral collaboration. Many of the members have been working together over a period of several years conducting applied research and collaborating on program and policy approaches to prevention, support and treatment related to HIV and AIDS. All of the team members were members of the team when the team was awarded a 1-year Ontario HIV Treatment Network Strategic Applied Research and Training (START) Development Grant in Prevention Research. This type of grant was awarded to multidisciplinary teams of community-based and/or academic researchers to develop community-based research in HIV prevention. For our team, the purpose of the START grant was to develop the infrastructure to design the present Phase II behavioural intervention trial to reduce HIV transmission risk behaviour among HIV+ MSM.

***Principal Investigators***

**Dr. Trevor A. Hart**, Assistant Professor of Psychology at Ryerson University, conducted studies at the Division of HIV/AIDS Prevention at the U.S. Centers for Disease Control and Prevention (CDC) examining psychological factors that predict HIV transmission behaviour among MSM in the Seropositive Urban Men's Study (SUMS) that contributed to the development of Seropositive Urban Men's Intervention Trial (SUMIT), one of the only randomized control trials for HIV+ MSM. Dr. Hart was trained by the NIH Summer Institute on Randomized Controlled Trials Using Behavioural Interventions. He holds a CIHR New Investigator Award in HIV Research and is funded by CIHR to examine how psychological distress increases sexual risk behaviour among HIV+ and HIV- MSM. He is a member of the Ontario Ethnoracial MSM Research Working Group. Dr. Hart is also a clinical health psychologist who conducts both psychotherapy and health improvement interventions with MSM and HIV+ populations using cognitive-behavioural and motivational enhancement counseling approaches.

**Dr. Barry D. Adam**, University Professor of Sociology at the University of Windsor, has carried out several major community-based research studies in collaboration with the AIDS Committee of Toronto on HIV risk taking among MSM, including qualitative and quantitative analyses of broad-based populations of HIV+ and HIV-negative MSM, high-risk men, couples, and bareback circuits, as well as research into family, relationship, work, and adherence issues faced by people living with HIV. He is currently Senior Scientist and Director of Prevention Research at the Ontario HIV Treatment Network and serves on the Ontario Gay Men's HIV Prevention Strategy Provincial Advisory Body, and the Ontario Advisory Committee on HIV and AIDS (OACHA) to MOHLTC.

***Co-Investigators***

**Herbert Co** is a Sexual Health Educator and Community Grants Officer for the Drug Prevention Community Investment Program at Toronto Public Health. Previous to this, he was the volunteer coordinator of the Asian Community AIDS Services. His involvement in HIV/AIDS includes began a peer trainer for the Ethnoracial Treatment Support Network, HIV/AIDS Treatment Counselor at CATIE, and moderator for the poz prevention focus group for East and Southeast Asians living with HIV/AIDS. He is a member of the Poz Prevention Working Group.

**David Hoe** worked for 13 years as a senior policy advisor on HIV/AIDS with the Government of Canada, and has been a co-chair of OACHA, a member of the Steering Committee of the Community-Based Research Evaluation Unit at McMaster University, and Executive Director of the AIDS Committee of Ottawa. He chaired the development of Ontario’s Strategy for HIV/AIDS to 2008 and received the Red Ribbon Award of the Canadian Association for HIV Research in 2005 for significant contributions to advancing research in the field of HIV/AIDS in Canada.

**Bob Leahy** is a person living with HIV/AIDS and a member of the Poz Prevention Working Group. He is a past chair and current secretary to the board of the Peterborough AIDS Resource Network (PARN). He has served on the board and has been treasurer of the Ontario HIV Treatment Network and has extensive experience with community-based initiatives supporting people living with HIV/AIDS.

**Dr. Mona Loutfy** has extensive community based research experience and heads the [Women and HIV Research Program](http://www.womensresearch.ca/programs/women_HIV.php) at Women’s College Research Institute. She also serves at Research Director at the [Maple Leaf Medical Clinic (MLMC)](http://www.mapleleafmedical.com/), a medical practice of 10 physicians (eight primary care physicians and two specialists) who specialize in HIV management. Dr. Loutfy is an Assistant Professor in the [Department of Medicine](http://www.deptmedicine.utoronto.ca/) and has medical appointments at Women's College Hospital, North York General Hospital, St. Michael's Hospital and a courtesy appointment at Sunnybrook Health Sciences Centre. In addition to her medical specialties, Dr. Loutfy also has a Masters in Public Health from the Harvard School of Public Health.

**Robert MacKay** is co-chair of the AIDS Bureau’s Poz Prevention Working Group and is currently a director on the national board of the Canadian AIDS Treatment Information Exchange (CATIE). He serves on the Council of The College of Chiropractors, having been appointed by the Lieutenant Governor. He is the past president of AIDS Thunder Bay and a member of their board for over 12 years. For six years he worked with the Ontario HIV Treatment Network to develop an electronic patient record to support observational research.

**Dr. Eleanor Maticka-Tyndale**, University Professor of Sociology at the University of Windsor, designed and conducted Comprehensive Dynamic Trials using mixed methods (interviewing, observation and surveys) to evaluate HIV prevention programs in Thailand and Kenya. The intervention designed and tested in Kenya is now being delivered in all primary schools in the country (>18,000) and is among the interventions in low-resource settings designated for packaging as one of the HAPPA programmes. Based on this work, she was invited to be a member of a WHO funded team where she was responsible for synthesizing the information available on evaluated community-based HIV prevention programming targeting youth in low income countries. She holds a Canada Research Chair in Social Justice and Sexual Health, is a fellow of the Society for the Scientific Study of Sexuality, and has been an active volunteer with ASOs in Montreal, Calgary and Windsor.

**John Maxwell** is Director of Special Projects with the AIDS Committee of Toronto. Prior to his appointment to this position, he was responsible for overseeing HIV prevention education programs at Canada's largest AIDS service organization. He is a member of the Ontario Gay Men's HIV Prevention Strategy Provincial Advisory Body and Poz Prevention Working Group, and has been involved in a number of community-based research projects on HIV risk among MSM. He received the 2007 Red Ribbon Award from the Canadian Association for HIV Research.

**James Murray** is a Senior Policy Analyst with the AIDS Bureau, Ontario Ministry of Health and Long-Term Care. In this capacity, James oversees the development of an Ontario-wide HIV prevention strategy for gay and bisexual men. Prior to working at the AIDS Bureau, James worked for seven years at the AIDS Committee of Toronto developing and delivering HIV prevention programs to gay and bisexual men in Toronto.

**4.2 Overview of the Study Design**

Written informed consent will be obtained prior to study enrolment including permission to complete study measures. Those who agree to enrol and meet inclusion criteria will be scheduled for a single assessment session to complete a demographics questionnaire, self-report measures, and a brief interview. Participants will complete 6 sessions of a small-group sexual health intervention to reduce sexual risk behaviour. Assessments will be conducted at the completion of each 6-session group, with follow-up assessments conducted at post-intervention and at 3 months following the end of each group.

**4.3 Sample**

Individuals will be eligible to participate in the intervention if they: (1) are HIV+ men who report UAI with a partner of HIV-negative or unknown HIV status in the past 3 months; (2) speak and understand English, (2) anticipate that they will be able to attend all workshops; and (3) are willing to participate in program monitoring and evaluation. Persons will be excluded if it is found that their ability to respond to study measures could be compromised by 1) central nervous system conditions (e.g., advanced HIV-associated dementia), 2) acute psychotic conditions, 3) acute mood dysregulation (e.g., manic states), and/or 4) debilitating physical conditions. For ethical reasons, all participants may withdraw from the research component of the study while still remaining in the intervention. Based on the literature, we anticipate that a number of participants will display psychological or other lifestyle characteristics that are strongly associated with sexual risk taking. They will be offered referral to counselling where appropriate; however, the decision to pursue such additional support will be left with the individual men and will not influence their eligibility to participate in the intervention. To avoid conflict of interest, participants who are given referrals will be referred to counselling with providers outside the study team. In addition, if a participant experiences distress at any time, referrals for counselling will be ready at hand. Intervention program facilitators will be trained in group facilitation and will be knowledgeable about referral sources for HIV+ group members who seek information or counselling, and the project will be housed at the AIDS Committee of Toronto which has HIV-related counselling and information resources.

**4.4 Sample Size Estimation**

For the quantitative data analyses, to examine changes in UAI with HIV-negative or unknown serostatus partners, random coefficient regression (also known as multilevel modeling) analyses will be conducted. The random regression model employs time as a within-subjects variable, and subject-specific intercepts and slopes for the time effect (i.e., trajectories) are summarized with mean and variance parameters. The analysis has several important advantages. It accounts for serial correlation within subjects, is relatively robust to randomly missing data, and can incorporate certain non-random missing data, especially those arising from drop-outs, without biasing model estimates. Following the approach for trajectory modeling outlined by Muthén & Muthén (2002), power analyses suggest an N=40 to detect a meaningful average change (i.e., medium effect size) in UAI with α=.05, and 1-β=.80. Given that the groups will have 6 persons each, with 7 groups, the final sample size will be N=42.

**4.5 Recruitment**

Our recent findings suggest that about half of HIV+ men are likely to have had unprotected sex with a partner who is HIV- or of unknown sero-status^75^. Recruitment will be done from a clustering of venues, identified in our research, where men who have unprotected sex most or all of the time are over-represented^4^. Our team has found these venues to be accessible and their participants to be interested in participation in research. We do not expect HIV+ MSM to be attracted to workshops billed as being only about HIV prevention, but rather to a program with a larger sexual health agenda developed through our previous studies. We will seek, where possible, to encourage the participation of men with diverse ethno-cultural backgrounds and will call on the experience of research team and PPWG members who are themselves HIV+ members of Asian, Latin American, and Caribbean communities to assist in this process.

Advertisements will be placed in newspapers and magazines serving the gay community (e.g., *Fab, Xtra*) and PHAs (e.g., *The Positive Side*). Flyers about the study will also be given for distribution by AIDS Service Organizations, such as the AIDS Committee of Toronto and ethnoracial-specific AIDS service organizations (e.g., African and Caribbean Council on HIV/AIDS members, Alliance for South Asian AIDS Prevention, Asian Community AIDS Services) in the Greater Toronto Area. All study flyers and advertisements will describe the nature of the study and will provide the phone number and email of the study coordinator. Interested participants will be invited to come to the study office in downtown Toronto to learn more. Both the study coordinator and our two HIV+ gay male facilitators will conduct recruitment. In this setting, participants will have an opportunity to ask questions and receive additional explanation before signing a consent form. The consent form will include an explanation of the study, risks and benefits of participation, the duration and type of participation, description of the procedures, contact persons for the research including the chairs of the University of Windsor and Ryerson University Research Ethics Boards, voluntary nature of participation, and the right to withdraw without penalty.

**4.6 Setting**

All data will be collected at the AIDS Committee of Toronto in downtown Toronto. This location is an ideal site for conducting the proposed study, given its convenient location near public transit and proximity to areas in which large numbers of HIV+ MSM reside, and its familiar location for many HIV+ MSM. The setting was also carefully chosen as a location in which the intervention would be delivered in the community, as the AIDS Committee of Toronto is the largest AIDS service organization in Canada. Our research team’s preliminary community capacity building and training study, funded by an Ontario HIV Treatment Network community-based prevention research START grant, has been housed at the AIDS Committee of Toronto, making the transition to work on the present study simple and feasible. Drs. Hart and Adam have extensive experience conducting research and interviewing research participants in a variety of community settings frequented by both HIV+ MSM and therefore are familiar with logistic factors involved in conducting research with these populations. Further, Dr. Hart is a registered clinical health psychologist with over 10 years experience counselling HIV+ MSM.

**4.7 Consent procedures**

Participants who contact the study office will be asked if they have an interest in participating in the study. If participants agree to participate, an appointment will be scheduled to conduct their assessment. At this time, the study will be explained and participants will be given an opportunity to ask questions and receive additional explanation before signing a consent form. Participants then will be asked to sign an informed consent form including an explanation of the study, risks and benefits of participation, the duration and type of participation, description of the procedures, contact person for the research including the chairs of the Ryerson University and University of Windsor Research Ethics Boards, voluntary nature of participation, and the right to withdraw without penalty.

**4.8 Potential risks**

Completion of the assessments and interventions are free of risks for physical harm. However, the assessment and intervention may raise issues that may be troublesome to some of the participants. Dr. Hart, the study coordinator, and the peer facilitators have previously been trained to deal with any emotional issues that may arise during the assessment and intervention. In addition, referral information (for counsellors, medical treatment or possibly other support or prevention information) will be made available to participants as needed. Research assistants will be supervised by a licensed clinical psychologist (Dr. Hart) who is experienced in working with both MSM and HIV patients. Further, the intervention is located at the AIDS Committee of Toronto, which has a large wealth of counselling and health resources for PHAs.

**4.8.1 Procedures to minimize risks**

Given the potentially sensitive nature of the information on sexual risk that may be gathered, we have a set of safeguards to minimize risks to participants that have worked well over a decade of similar kinds of research. Threats to confidentiality during the assessments will be minimized by using code numbers, not names, for assessments. At the end of data entry, data will be password-protected and backed-up on a master data file. All confidential material including code numbers and data will be kept in a locked office in locked file cabinets with limited access. The study will be submitted to the Ryerson and University of Windsor Research Ethics Boards for approval prior to assure risks are minimized. Threats to confidentiality during the intervention will be minimized by having all participants sign a form indicating that all information shared by members during the intervention will not be shared outside the group. This form is already available at the AIDS Committee of Toronto due to the many counselling and support groups that take place there. Participants also will be invited to use only their first names, or a pseudonym, as the participant prefers. Participants will not be aware of other participants’ code numbers for assessment.

**4.8.2 Risks versus benefits**

The primary risk for participation in the study is related to breaks in confidentiality. As noted above, several steps will be taken so that data will remain confidential both during and after the investigation. Another risk is the possibility that participants may become upset by the questions on the questionnaire. Although benefits to the participant are likely to be minimal, some participants may appreciate the study’s focus on mental health and its in-depth focus on what may actually be happening in sexual situations that may inhibit or promote healthy sexual behaviour.

**4.9 Monitoring and Evaluation of the Intervention**

**4.9.1. Comprehensive Dynamic Trials**

The framework of *continuous quality improvement design (CQID)*^76^ will guide the development of monitoring and evaluation for this project. CQID is particularly well suited to interventions that are grounded within communities and social networks, that must be able to accommodate diverse needs in a heterogeneous population, and that include goals of maximizing effectiveness and responsiveness to community needs. Monitoring and evaluation in CQID are designed to collect information not only on the targeted outcomes but also on mediating factors. Data are collected from multiple sources (e.g. participants, facilitators, participation records) using multiple methods. Analysis explores patterns of outcome and response to the program, tracing pathways of influence on the thinking, choices and actions of different groups of participants, thereby providing information about how different participants interpret and respond to the intervention based on their particular personal and social locations. Information about what works in various contexts and for various people is fed back into the intervention through the study team that has representation from diverse stakeholders in order to guide modifications for enhancing the intervention’s acceptability, responsiveness to the community, and effectiveness in achieving the desired objectives. CQID takes an intervention through several iterations with monitoring and evaluation informing modifications or retooling between iterations.

**4.9.2 Mixed Method Approach: Quantitative and Qualitative Data**

Quantitative data will be collected at the study site through a battery of self-report questionnaires and interviews. Facilitators will also maintain attendance records. Based on analysis of the structured assessments and surveys at each of the three data collection points, a selection of participants will also participate in face-to-face semi-structured interviews. Participants will be selected to represent a broad diversity of experiences related to UAI, intermediary factors, and responses to the intervention. It is estimated that approximately 10 of the total sample size of N=42 participants will be randomly selected to be recruited for interviews at each of the 2 data collection points (not necessarily the same individuals at each timepoint). Interview participants will be asked to provide greater detail related to their responses to the survey, exploring in depth their opinions about the intervention they have completed.

Participants will complete brief assessments at the end of each workshop, responding to approximately 5 questions about that workshop: (a) What did you like the best and why? (b) What did you like the least and why? (c) What do you think is most useful to you? (d) Would you recommend this session to a friend? (e) Are there some people for whom this session would be particularly useful? Please describe them. Group facilitators will compile notes at the close of each session answering the following questions: (a) What went particularly well? Why? (b) What did not go as well as you had hoped or anticipated? Why? (c) What was missing that should be considered for inclusion in the future? (d) How would you assess the group dynamics? (e) What unsolicited comments were made to you about this session by participants? (f) What else do you want to say about this session?

The quantitative and qualitative data collected during the monitoring and evaluation will be analyzed both independently and in tandem, with results of each analysis informing the other. In-depth interviews will be used to further elaborate and clarify the quantitative results and will also lead to potentially new questions for quantitative analysis. Given the nature and size of the sample (42 participants), the Phase II nature of the intervention, and consistent with the CDT design, the goal of the monitoring and evaluation will be provision of information from diverse data sources that contributes to improving the intervention for our future studies.

**4.9.3 Quantitative Measures**

The quantitative measures used in this study have been demonstrated to possess good reliability and validity, as well as documented associations with risky sexual behaviour^23,51,52,59,60^. It is anticipated that the entire protocol will take approximately 50 minutes to complete at each timepoint: baseline, post-intervention, and 3 months after the end of the intervention.

**Demographics Questionnaire.** Participants will complete a demographics questionnaire assessing age, ethnic/racial background, religious affiliation, educational level, living situation, marital/partner status, socioeconomic status, and sexual orientation.

**Primary Endpoint Variable: Unprotected Sexual Behaviour.** Participants will be asked to indicate their frequency of unprotected anal intercourse with HIV-negative or unknown HIV status partners in the past 3 months.

**Secondary Endpoints.** Secondary endpoints will also examine protected and unprotected 1) insertive and receptive anal intercourse with men, 2) anal and/or intercourse with women with and without a condom in the previous 3 months with partners who were HIV+, partners who were HIV-negative, and partners of unknown serostatus. Second, participants will be asked to indicate their number of HIV+ partners, HIV-negative partners, and partners of unknown serostatus in the previous 3 months. Third, participants will be asked if they had insertive or receptive anal intercourse or vaginal intercourse with their last sex partner, as well as their last partner’s serostatus.

**Potential Theoretical Mediators.** Per the IMB model, Information, Motivation, and Behavioural Skills will be assessed to examine the relative effects of each component on UAI with non-HIV+ partners. The HIV-Knowledge Questionnaire-18^76^ will be used to assess knowledge of basic HIV information. Questions will also ask about knowledge of very recent data, such as the PHAC’s statement on the Swiss Cohort Study^77^, and knowledge of criminalization of unprotected sex for HIV+ individuals (CITES). Participants’ motivations to perform 8 risk-reduction actions will be assessed per recent research assessing the IMB model^60^. Examples of items include intentions to keep condoms nearby, to use a condom with a new sexual partner, and to use a condom when drunk or high on a substance. Self-efficacy for behavioural skills in risk reduction will be assessed using a 6-item measure^60^. Items include “I am certain I can use a condom with my main sex partner.” Participants will also be asked about whether they have engaged in 5 risk reduction behavioural skills over the past month, such as refusing to have unsafe sex and guiding sex toward safer sex behaviour.

**Additional Measures.** Due to data implicating substance use in unprotected intercourse among MSM**^38^**.**^78-81^**, participants will indicate how many times they have been intoxicated when drinking alcohol or used an injection or non-injection substance in the past 3 months. To assess for possible confounding due to social desirability reporting bias on sensitive behaviours such as sexual behaviour and drug use, participants will complete the Marlowe Crowne Social Desirability Scale - Short Form^81^. If any of these variables is associated with unprotected sexual behaviour, they will be examined as potential other mediators in analyses examining the effect of the intervention on unprotected anal intercourse with HIV-negative or unknown HIV status partners. CD4 count and viral load will be requested through self-report for the present study. These measures will serve as medical indices of severity of HIV illness in our HIV+ sample.

**4.10 Stages of the Study**

**4.10.1 Research Stage I – Study Initiation**

In Month 1, the study coordinator and 2 group facilitators from our previous Ontario HIV Treatment Network START grant on Poz Prevention will be re-hired. The project timeline can be found in Appendix 2. Both facilitators are HIV+ gay men with extensive experience with group facilitation. Through the START grant, our facilitators have been trained by Dr. Tim Guimond of the Centre for Addiction and Mental Health in motivational enhancement counselling techniques for HIV prevention. Program staff will be trained in study participant recruitment, administration of self-report measures, and data entry. Procedures for all aspects of project management will be established and implemented during the first 3 months of the project. A weekly planning meeting of staff and investigators will be initiated to begin development of project infrastructure. Staff will regularly coordinate with Drs. Hart and Adam to ensure efficiency in participant recruitment. A protocol manual will be created for conducting all aspects of the study, including detailed information on participant eligibility criteria, recruitment procedures, informed consent procedures, administration of research measures, and data management plans. Standardized project forms also will be created (e.g., protocol checklists). In addition, facilitators will be trained that if a participant experiences distress at any time, referrals for counselling will be ready at hand. Intervention program facilitators will be trained in group facilitation and will be knowledgeable about referral sources for HIV+ group members who seek information or counselling, and the project will be housed at the AIDS Committee of Toronto which has HIV-related counselling and information resources. Program delivery staff/trainees will be supervised by Dr. Hart.

**4.10.2 Research Stage II – Data Collection**

Data collection will begin in month 4 of Year 1 and will continue through month 6 of Year 2 of the study. Persons in the study who contact the study office will be informed about the opportunity to participate in the study while waiting for their appointments. Specifically, they will be told that the study is involves a series of workshops intended to improve sexual health among HIV+ MSM, that participation entails responding to several questionnaires and a brief interview at a baseline assessment taking about 120 minutes to complete, a series of 6 workshops of 90 minutes each, a post-intervention assessment of 60 minutes, and a 3-month follow-up of 120 minutes. They will also be told that $30 per hour will be provided in exchange for participation. Drs. Adam and Hart have found these recruitment procedures to be both unobtrusive and effective in other larger scale studies of HIV+ MSM.

Upon obtaining informed consent, quantitative measures will be administered in questionnaire format. Upon completion of the protocol, participants will be debriefed and given the opportunity to ask any questions. Participants will then be remunerated at $30 per hour. Qualitative interviews will explore in greater depth the evaluative questions in 4.9.2 above and also the degree to which the workshops resonated with participants’ everyday lives by asking about decision-making in recent sexual encounters.

**4.10.3 Research Stage III – Data Entry and Analysis**

The study coordinator will oversee data management procedures established in study stage I once data collection has begun. Data entry and analysis will proceed until month 9 of Year 2 of the study. Statistical analysis will be conducted under the guidance of the quantitative psychologist of the study team, Dr. David Flora, who is well-trained in analyses of behavioural intervention data. Completed protocols will be stored in a locked file cabinet and only assigned participant identification numbers will appear on completed assessment materials to protect confidentiality. A data file will be created using SPSS or STATA into which coded data will be entered. If the primary endpoint is not normally distributed, it will be transformed using the formula [log_10_ (x +1)]^82^ to avoid violations of assumptions of normality in parametric analyses. If this does not sufficiently correct for positive skew or kurtosis, data will be dichotomized per standard convention in the HIV behavioural literature into presence or absence of unprotected sex with non-HIV+ partners in the past 3 months.

**4.11 Research Stage IV: Disseminate Findings,Write Proposal for RCT**

After a thorough assessment of the strengths and weaknesses of the proposed intervention, together with feedback from stakeholder communities, a manual describing the intervention as set within a consultative model will be developed and work will begin on the development of a proposal for delivery and testing of the modified intervention in Toronto and other smaller sites. The training resources and findings from this project will enter into the larger Ontario Gay Men’s HIV Prevention Strategy.

A key problem with many interventions created through research is the lack of consultation with community agencies that will actually administer the intervention upon completion of research activities^75^. Thus, in the final phase, the research team and the rest of the Poz Prevention Working Group will return with results to stakeholder communities for discussion on how to integrate the future RCT into community work, and organize results to present to community forums, policy makers, the Ontario AIDS Network, the Ontario HIV Treatment Network and the Canadian Association of HIV Research conferences, and potentially international conferences. With consultation with agencies that will help to deliver the intervention, the primary investigators will then refine the intervention manual to reflect the way the intervention was delivered in the present study. The team will then design and implement a RCT to test the efficacy of the intervention in Toronto and other smaller sites throughout Canada. The intervention evaluated in this Phase II design can be compared to both treatment-as-usual and social cognitive theory-based interventions that were efficacious among general samples of PHAs but that do not specifically address barebacking among MSM^48,49^.

**5.0. relevance and potential impact**

Despite the need for interventions that address increasing HIV diagnoses among MSM, higher rates of UAI among HIV+ versus HIV-negative MSM, and the barebacking phenomenon among MSM, there is a lack of interventions that specifically focus on HIV+ MSM, many of whom may not be motivated to use condoms during anal intercourse. The primary objective of the present study is to evaluate a small-group HIV prevention program for HIV+ MSM using motivational enhancement counselling to reduce sexual risk behaviour. An effective HIV prevention intervention for HIV+ MSM has the potential to make a significant difference in epidemiological trends in HIV in Canada.

The proposed study will provide data on the effect size of a small group-based motivational enhancement counselling behavioural intervention for HIV+ MSM. Although behavioural intervention research among primarily American samples of non-HIV+ MSM and HIV+ persons who are not exclusively MSM has documented effect sizes for HIV prevention interventions^41^, none have found a significant effect of an HIV prevention behavioural intervention for HIV+ MSM in Canada, nor have any studies examined the effect of a small-group based intervention using motivational enhancement counselling for HIV+ MSM. Armed with the data from the present study demonstrating the effect size of the intervention, the study team will then design and implement a randomized controlled trial to examine the efficacy of the intervention in Toronto and other sites serving HIV+ MSM in Canada.

Second, the present study would be an excellent example of the potential benefits of conducting a conducting a Phase II intervention trial in a community setting. The present study is one of the first to link clinical intervention research methods and community-based research in North America. Many empirically tested HIV prevention interventions have not been guided by the spirit of community collaboration, which may have limited community preparedness to roll out these interventions in the United States^83^. The present study avoids this pitfall by choosing a community-based approach to the testing of HIV prevention interventions. If the randomized controlled trial following the proposed study is found to be efficacious in reducing sexual risk behaviour, the study intervention has an increased likelihood of being sustainable at the AIDS Committee of Toronto, the largest AIDS service organization in Canada. Given that the AIDS Committee of Toronto is also well-established and respected across Canada, the intervention may also have an increased likelihood of being taken up as an intervention at other AIDS service organizations in Canada. Even more importantly, one of our team members, James Murray, is also a representative from the Ontario AIDS Bureau, assuring the intervention will have already have the base of a province-wide forum. Further, the present study, which links current established clinical mental health techniques^54^ with sustainable, community-based HIV prevention work, is highly innovative. It is a goal of the researchers for Canada to be known not only for its advances in HIV medical interventions and treatments, but also for its advances in community-based HIV behavioural interventions.

**References**

| 1. | Remis R, Swantee C, Schiedel L, Liu J. *Report on HIV/AIDS in Ontario 2006*. Toronto: Ontario HIV Epidemiolgoic Monitoring Unit. 2008. |
| --- | --- |
| 2. | Remis R., Liu J. *The HIV epidemic in Ontario*. Available at: http://www.phs.utoronto.ca/ohemu/doc/Epi2006%20Remis%20Dec06.pdf. Accessed April 7, 2007. |
| 3. | Hart TA, James CA, Hagan C, Boucher E. HIV Optimism and High Risk Sexual Behaviour in Two Cohorts of Men Who Have Sex with Men in Toronto, Canada, 2005-2007. *Can J Infect Dis Med Microbiol.* 2008;19:Suppl A. |
| 4. | Adam BD, Husbands W, Murray J, Maxwell J. Circuits, networks, and HIV risk management *AIDS Educ Prev.* 2008;20(5):420-435. |
| 5. | Adam BD, Husbands W, Murray J, Maxwell J. *Risk Management in Circuits of Gay and Bisexual Men*. Toronto: AIDS Committee of Toronto. 2007. |
| 6. | Adam BD, Husbands W, Murray J, Maxwell J. Silence, assent, and HIV risk. *Cult Health Sex.* 2009 (in press) |
| 7. | Gordon CM, Stall R, Cheever LW. Prevention interventions with persons living with HIV/AIDS. *J Acquir Immune Defic Syndr.* 2004;37(2):S53-S57. |
| 8. | Wingood G, DiClemente R, Mikhail I, et al. A randomized controlled trial to reduce HIV transmission risk behaviors and sexually transmitted diesases among women living with HIV. *J Acquir Immune Defic Syndr*. 2004;37(2):S58-S67. |
| 9. | Semple S, Patterson T, Grant I. Partner Type and Sexual Risk Behavior Among HIV Positive Gay and Bisexual Men. *AIDS Educ Prev.* 2000;12(4):340-56. |
| 10. | Rhodes T, Cusick L. Accounting for Unprotected Sex. *Soc Sci Med.* 2002;55:222. |
| 11. | Richters J, Hendry O, Kippax S. When Safe Sex Isn’t Safe. *Cult Health Sex.* 2003;5(1). |
| 12. | Gorbach PM, Galea JT, Amani B, Shin A, Celum C, Kerndt P, Golden MR. Don’t Ask, Don’t Tell. *Sex Transm Infect.* 2004;80:516 |
| 13. | Smith, Grierson A, Wain D, Pitts M, Pattison P. Associations Between the Sexual Behaviour of Men Who Have Sex with Men and the Structure and Composition of Their Social Networks. *Sex Transm Infect* 2004:80:455-58. |
| 14. | Adam BD, Husbands W, Murray J, Maxwell J. Risk construction in the reinfection discourses of HIV-positive men. *Health Risk Soc*. 2005;7(1):63-71. |
| 15. | Adam BD. Constructing the neoliberal sexual actor. *Cult Health Sex.* 2005;*7*(4):333-346. |
| 16. | Larkins S, Reback CJ, Shoptaw S, Veniegas R. Methamphetamine Dependent Gay Men’s Disclosure of Their HIV Status to Sexual Partners. *AIDS Care.* 2005;17(4):526. |
| 17. | O’Leary A. Guessing Games. In: Halkitis P, Gómez C, Wolitski R. eds. *HIV+ Sex*. Washington, DC: American Psychological Association; 2005:121-32. |
| 18. | Stirratt, M. I Have Something to Tell You. In: Halkitis P, Gómez C, Wolitski R, eds. *HIV+ Sex* Washington, DC: American Psychological Association; 2005:101-19. |
| 19. | Körner H, Ellard J, Hendry O, Kippax S, Grulich A, Hodge S. *Taking post-exposure prophylaxis: Managing risk, reclaiming control* (Monograph 5/2003). Sydney: National Centre in HIV Social Research, The University of New South Wales. 2003 |
| 20. | Van de Ven P, Mao L, Fogarty A, et al. Undetectable viral load is associated with sexual risk taking in HIV serodiscordant gay couples in Sydney. *AIDS*. 2005;19:179-184. |
| 21. | Niccolai LM, Farley TA, Ayoub MA, Magnus M, Kissinger PJ. Positive, Negative, Unknown: Assumptions of HIV Status Among HIV-Positive Men Who Have Sex With Men. *AIDS Educ Prev*. 2006;18(2):139-149. |
| 22. | Adam BD, Husbands W, Murray J, Maxwell J. AIDS optimism, condom fatigue, or self esteem? *J Sex Res.* 2005;42(3):238-248. |
| 23. | Fisher J, Fisher W. *Contributions of the information-motivation-behavioral skills model to HIV prevention.* Presented at the International AIDS Conference, Geneva. 1998. |
| 24. | Grulich AE, Prestage GP, Kippax SC, Crawford JM, Van de Ven PG. “HIV Serostatus of Sexual Partners of HIV-Positive and HIV-Negative Homosexual Men in Sydney.” *AIDS.* 1998:12(18):2508. |
| 25. | Chen S, Gibson S, Wide D, McFarland W. Unprotected Anal Intercourse Between Potentially HIV-Serodiscordant Men Who Have Sex with Men, San Francisco. *J Acquir Immune Defic Syndr.* 2003;33:166-70. |
| 26. | Dodds JP, Mercey DE, Parry JV, Johnson AM. *Recent Trends in Sexual Behaviour Amongst Men Who Have Sex with Men.* Presented at the International AIDS Conference, August, Toronto. 2006. |
| 27. | Elford J, Bolding G, Sherr L. High risk sexual behaviour increases among London gay men between 1998-2001: what is the role of HIV optimism? *AIDS*. 2002;16:1537-1544. |
| 28. | Mansergh G, Marks G, Colfax G, Guzman R, Rader M, Buchbinder S. 'Barebacking’ in a Diverse Sample of Men Who Have Sex with Men. *AIDS*. 2002;16:653-59. |
| 29. | Rogers G, Curry M, Oddy J, Pratt N, Beilby J, Wilkinson D. Depressive Disorders and Unprotected Casual Anal Sex Among Australian Homosexually Active Men in Primary Care.” *HIV Med*. 2003;4:271-75. |
| 30. | Elford J, Bolding G, Davis M, Sherr L, Hart G. Trends in Sexual Behaviour Among London Homosexual Men 1998-2003.” *Sex Transm Infect.* 2004;80:451-54. |
| 31. | Hospers H, Kok G, Harterink P,de Zwart O. A New Meeting Place. *AIDS.* 2005;19:1097-1101. |
| 32. | Morin S, Steward W, Charlebois E, et al. Predicting HIV Transmission Risk Among HIV-Infected Men Who Have Sex with Men. *J Acquir Immune Defic Syndr.* 2005;40(20):226-35. |
| 33. | Whittier D, St Lawrence J, Seeley S.Sexual Risk Behavior of Men Who Have Sex with Men. *Arch Sex Behav.* 2005;34(1):95-102. |
| 34. | Cox J, Lambert G, Alary M, et al. *Enquête sur le VIH, les Hépatites Virales et les ITS Ainsi Que sur les Comportements À Risques Associés.* Presented at the M-Track HIV Surveillance System Workshop. Ottawa: Public Health Agency of Canada. 2006. |
| 35. | Frankis JS, Flowers P. Cruising for Sex. *AIDS Care.* 2006; 18(1):54-59. |
| 36. | Peterson J, Bakeman R. Impact of Beliefs About HIV Treatment and Peer Condom Norms on Risky Sexual Behavior Among Gay and Bisexual Men. *J Community Psychol.* 2006*;*34(1):37-46. |
| 37. | Dodds JP, Mercey DE, Parry JV, Johnson AM. *Recent Trends in Sexual Behaviour Amongst Men Who Have Sex with Men.* Presented at the International AIDS Conference, August, Toronto. 2006. |
| 38. | Myers T, Aguinaldo JP, Dakers D, Fischer B, Bullock S, Millson P, Calzavara L. How Drug Using Men Who Have Sex with Men Account for Substance Use During Sexual Behaviours.” *Addiction Res Theor.* 2004;12(3):213-29. |
| 39. | Adam BD. Infectious behaviour: Imputing subjectivity to HIV transmission. *Soc Theor Health.* 2006:4:168-179. |
| 40. | Johnson W, Holtgrave D, McClellan W, et al. HIV intervention research for men who have sex with men. *AIDS Educ Prev*. 2005:17(6):568-589. |
| 41. | Johnson WD. Behavioral interventions to reduce risk for sexual transmission of HIV among men who have sex with men (review). *Cochrane Collaboration*. 2008(3). |
| 42. | Albarracin D, Gillette JC, Earl AN, Glasman LR, Durantini MR, Ho M. A test of major assumptions about behavior change. *Psychol Bull.* 2005;131(6):856-897. |
| 43. | Kalichman SC, Rompa D, Cage M, DiFonzo K, Simpson D, Austin J, Luke W, Buckles J, Kyomugrsha F, Benotsch E, Pinkerton S, Graham J. Effectiveness of an Intervention to Reduce HIV Transmission Risks in HIV Positive Persons. *Am J Prev Med*. 2001;21, 84-92. |
| 44. | Roffman R, Stephens R, Curtin L, et al. Relapse prevention as an interventive model for HIV risk reduction in gay and bisexual men. *AIDS Educ Prev*. 1998:10(1):1-18. |
| 45. | Carballo-Diéguez A, Dolezal C, Leu C, et al. A randomized controlled trial to test an HIV-prevention intervention for Latino gay and bisexual men. *AIDS Care*. 2005;17(3);314-328. |
| 46. | Conner R, Takahashi L, Ortiz E. et al. The SOLAAR HIV prevention program for gay and bisexual Latino men. *AIDS Educ Prev*. 2005;17(4);361-374. |
| 47. | Kok G. Targeted prevention for people with HIV/AIDS*. Patient Educ Counsel.* 1999:*36:*239-246 |
| 48. | Kalichman S, Rompa D, Cage M. Group intervention to reduce HIV transmission risk behavior among persons living with HIV/AIDS. *Behav Modif*. 2005:29(2):256-285. |
| 49. | Patterson TL, Shaw WS, Semple SJ. Reducing the sexual risk behaviors of HIV+ individuals: outcome of a randomized controlled trial. *Ann Behav Med*. 2003;25:137-145. |
| 50. | Wolitski R, Parsons J, Gómez C, Purcell D, Hoff C, Halkitis P. et al. Prevention with gay and bisexual men living with HIV. *AIDS*. 2005;19(Supplement 1);S1-S11. |
| 51. | Fisher JD, Fisher WA. Theoretical approaches to individual-level change. In Peterson J, DiClemente R, eds. *HIV Prevention Handbook*. New York: Kluwer Academic/Plenum Press. 2002;3-55. |
| 52. | Fisher J, Fisher W, Bryan A, Misovich S. Information-motivation-behavioral skills model-based HIV risk behavior change intervention for inner-city high school youth. *Health Psychol.* 2002:21(2), 177-186. |
| 53. | Shernoff M. *Without Condoms.* New York: Routledge; 2006. |
| 54. | Rollnick S, Miller W, Butler C. *Motivational Interviewing in Health Care: Helping Patients Change Behavior (Applications of Motivational Interviewing): Helping Patients Change Behavior.* New York: Guilford; 2008. |
| 55. | Miller W, Rollnick S. *Motivational Interviewing.* Second Edition. New York: Guilford; 2002. |
| 56. | Rutledge S. Single-session motivational enhancement counseling to support change toward reduction of HIV transmission by HIV positive persons. *Arch Sex Behav.* 2007:36:313-319. |
| 57. | Public Health Agency of Canada. Canadian Guidelines for Sexual Health Education. Available online at: http://www.phac-aspc.gc.ca/publicat/cgshe-ldnemss/cgshe_toc.htm. Accessed October 20, 2008. |
| 58. | Carey M, Carey K. Behavioral risks for HIV infection among adults with a severe and persistent mental illness. *Community Ment Health J.* 1997;33(2), 133. |
| 59. | Fisher J, Cornman D, Osborn C, Amico KR, Fisher W, Friedland G. Clinician-initiated HIV risk reduction intervention for HIV-positive persons. *J Acquir Immune Defic Syndr*. 2004:7(Supplement2):S78-S87. |
| 60. | Kalichman S, Cain D, Weinhardt L et al. Experimental Components of Analysis of Brief Theory Based HIV/AIDS Risk-Reduction Counseling for Sexually Transmitted Infection Patients. *Health Psychol*. 2005;24;2:198-208. |
| 61. | Kalichman SC, Picciano JF; Roffman RA. A Motivation to reduce HIV risk behaviors in the context of the Information, Motivation and Behavioral Skills (IMB) model of HIV prevention. *J Health Psychol.* 2008;13(5):680-9. |
| 62. | [Picciano JF, Roffman RA, Kalichman S, Rutledge S, Berghuis J. A Telephone Based Brief Intervention Using Motivational Enhancement to Facilitate HIV Risk Reduction Among MSM: A Pilot Study.](../../../../C:/Users/OHTN%20Grant/Narratives/Final%20Citations_CIHR.xls#RANGE!ContactOfAuthor1%23RANGE!ContactOfAuthor1) *AIDS Behav. 2001:5,3.* |
| 63. | | Bancroft J, Janssen E, Strong D, Carnes L, Long JS Sexual risk taking in gay men: The relevance of sexual arousability, mood, and sensation seeking. *Arch Sex Behav*. 2003;32:555-572. | | --- | |
| 64. | Lammers M, Davidovich U, Prins M, Stolte I. *Condom induced erectile dysfunction (COINED): a unique predictor of deliberate sexual risk.* International AIDS Conference, Mexico City. 2008. |
| 65. | Adam BD, Elliott R, Husbands W, Murray J, Maxwell J. Effects of the criminalization of HIV transmission in Cuerrier on men reporting unprotected sex with men” *Can J Law Soc.* Forthcoming. |
| 66. | Prochaska J, DiClemente C. *S*tages and Processes of Self-Change of Smoking: Toward an Integrative Model of Change*. J Consult Clin Psychol.* 1983;51:3:390-995*.* |
| 67. | Wolitski R, Parsons J, Gomez C*.* Prevention With HIV-Seropositive Men Who Have Sex With Men. *J Acquir Immune Defic Syndr*. 2004:S104. |
| 68. | Fernandez MI, Perrino T, Collazo JB et al. Surfing New Territory. *J of Urban Health*. 2005;82;1:S79-S88. |
| 69. | Halkitis P, Wilton L. *Barebacking*. Binghamton, NY:Haworth Press. Halkitis and Drescher. 2005. |
| 70. | O'Leary A, Hoff C, Purcell D, et al. What happened in the SUMIT trial? *AIDS*. 2005:19(Supplement 1):S111-S121. |
| 71. | Wolitski RJ, Parsons JT, Go´mez CA, Purcell DW, Halkitis PN, and the SUMIT Study Team. Prevention with gay and bisexual men living with HIV: rationale and methods of the Seropositive Urban Men’s Intervention Trial. *AIDS.* 2005;19:Suppl1:S1–S11. |
| 72. | Nolan RP, Upshur R, Lynn H, Crichton RT, Stewart DE, Alter DA, Harvey PJ, Grace SL, Corace K, Barry-Bianchi SM. Therapeutic benefit of a standardized telehealth program in the community outreach heart health and risk reduction trial (COHRT): A randomized controlled trial. *European Journal of Cardiovascular Prevention and Rehabilitation.* EuroPrevent Congress Abstracts. May 2008;15:Supplement1pg.S32. |
| 73. | Friedman LM, Furberg CD, DeMets DL. *Fundamentals of Clinical Trials*. New York, NY: Springer Verlag; 1998:284-322. |
| 74. | Hart T, James C, Myers J, Roberts K. *HAART-Related Beliefs and Un-protected Anal Intercourse with Serodiscordant or Unknown HIV status Partners in a Canadian Sample of Men Who Have Sex with Men.* Presented at the International AIDS Conference. 2006. |
| 75. | Rapkin B, Trickett E. Comprehensive dynamic trial designs for behavioural prevention research with communities. In Trickett E, Pequegnat W, eds. *Community Interventions and AIDS.* New York: Oxford University Press. 2005:252-269. |
| 76. | Carey MP, Schroder KEE. Development and psychometric evaluation of the brief HIV knowledge questionnaire (HIV-KQ-18). *AIDS Educ Prev.* 2002;14:174-184. |
| 77. | [Canadian AIDS Treatment Information Exchange (CATIE). CATIE-News: Bite-sized HIV/AIDS treatment news bulletins: Swiss guidelines take a troubling turn. Available online from: http://www.catie.ca/catienews.nsf/news/A2EFD4077BD44FB285257405005EE5EF?OpenDocument. Accessed on October 20, 2008.](http://www.catie.ca/catienews.nsf/news/A2EFD4077BD44FB285257405005EE5EF?OpenDocument) |
| 78. | Bimbi D, Nanin J, Parsons J, Vicioso K, Missildine W, Frost D. Assessing Gay and Bisexual Men's Outcome Expectancies For Sexual Risk Unders the Influence of Alcohol or Drugs. *Subst Use Misuse.* 2006:41: |
| 79. | Halkitis PN, Parsons JT, Stirratt MJ. A Double Epidemic: Crystal Methamphetamine Drug Use in Relation to HIV Transmission Among Gay Men. *J Homosex*. 2001;41;2:17-35. |
| 80. | Halkitis PN, Shrem MT, Martin FW. Sexual Behavior Patters of Methamphetamine-Using Gay and Bisexual Men. *Subst Use Misuse.* 2005;40:703-719. |
| 81. | Reynolds WM. Development of reliable and valid short forms of the Marlowe-Crowne Social Desirability Scale. *J Clinical Psych.* 1982;38:1;119-125. |
| 82. | Kirk RE. *Experimental design: Procedures for the behavioral sciences.* Belmont, CA: Wadsworth. 1968. |
| 83. | Dworkin S, Pinto RM, Hunter J, Rapkin B, Remien RH. Keeping the Spirit of Community Partnerships Alive in the Scale Up of HIV/AIDS Prevention: Critical Reflections on the Roll Out of DEBI (Diffusion of Effective Behavioral Interventions). *Am J Community Psychol*. 2008;42:1-2:51-59. |
